# Bioassay-guided isolation of sesamin and fargesin from the hydroalcoholic stem extract of *Zanthoxylum armatum* DC. inhibited inflammation in CpG-stimulated conventional type 1 dendritic cells

**DOI:** 10.3389/fphar.2025.1687789

**Published:** 2025-11-07

**Authors:** Ningthoujam Indrajit Singh, Chayan Mukherjee, Jerina Soibam, Wangkheimayum Marjit Singh, Naresh Chandra Bal, Sunil Kumar Raghav, Chingakham Brajakishor Singh

**Affiliations:** 1 Plant Bioresources Division, Institute of Bioresources and Sustainable Development (IBSD), Imphal, Manipur, India; 2 School of Biotechnology, Kalinga Institute of Industrial Technology (KIIT), Bhubaneswar, Odisha, India; 3 Institute of Life Sciences, Bhubaneswar, Odisha, India; 4 Department of Chemistry, Ghanapriya Women’s College, Dhanamanjuri University, Imphal, Manipur, India

**Keywords:** *Zanthoxylum armatum* DC., sesamin, fargesin, intracellular IL12p40/70, CD80

## Abstract

**Introduction:**

*Zanthoxylum armatum *DC. is renowned for its medicinal values. All the plant parts have been used to treat tooth- and gum-related problems, gastro-intestinal problems, inflammation, rheumatism, and pain by the indigenous people of Nepal, India, China, and other South East Asian countries. Bioassay-guided isolation of active compounds from medicinal plants is recognized as a promising approach for the discovery of novel drug candidates. The objective of this study was to examine the main constituents of *Zanthoxylum armatum* DC. stems through bio-guided isolation and to explore their anti-inflammatory potential.

**Method:**

Sequential fractions were prepared from the hydromethanolic stem extract of *Z. armatum* DC. Afterward, bioassay-guided isolation was conducted using a combination of column chromatography, heat-induced hemolysis inhibition assay, and albumin denaturation inhibition assay. The structures of the isolated compounds were elucidated through single crystal XRD and NMR. The anti-inflammatory activity of the compounds was evaluated *in vitro* by measuring the expression levels of IL12 and CD80 using flow cytometry.

**Results:**

Sequential ethyl acetate fraction showed the highest protein anti-denaturation and membrane stabilization activities. Afterward, sesamin and fargesin were isolated from the sequential ethyl acetate fraction. Both of them showed activity against IL12 production by conventional type 1 dendritic cells. Moreover, fargesin significantly inhibited the expression of CD80.

**Conclusion:**

The results identified bioactive compounds with potential against the pro-inflammatory dendritic cells for the first time. The findings justified the traditional use of *Z. armatum* DC. as an anti-inflammatory agent.

## 1 Introduction

Inflammation is a complex biophysiological process that protects living tissues from harmful insults of inflammatory agents such as pathogens, toxins, and damaged cells (Medzhitov, 2021). The clinical signs of these nonspecific immune reactions include local redness, swelling, heat, pain, fever, and occasional loss of function. Controlled, short-term inflammation plays a protective role in restoring tissue homeostasis. However, a shift from an acute to a chronic response can lead to the breakdown of immune tolerance, contributing to the development and progression of a wide range of diseases, including inflammatory bowel disease, rheumatoid arthritis, atopic dermatitis, psoriasis, and asthma (Wu, 2023). Steroidal anti-inflammatory drugs (SAIDs) and nonsteroidal anti-inflammatory drugs (NSAIDs) are commonly prescribed for inflammatory diseases. However, SAIDs may cause several side effects, including hormonal imbalance, unexpected weight gain with increased appetite, and immunodeficiency. Similarly, NSAIDs can lead to adverse outcomes, such as stomach ulcerations, hepatotoxicity, allergic reactions, and metabolic disorders (Panchal and Sabina, 2023; Yatoo et al., 2018). Pro-inflammatory mediators—such as cytokines, nitric oxide, prostaglandins, reactive oxygen species, and neutrophil-derived free radicals—are cytotoxic not only to pathogens but also to host cells. Their overproduction during inflammation can cause damage to host tissues by attacking macromolecules, including lipid membrane peroxidation, increasing vascular permeability, promoting vasodilation, and damaging endothelial tissues, all of which play a significant role in various inflammatory diseases (Amro and Daniel, 2022; Medzhitov, 2021; Anoop and Bindu, 2015). As inflammatory disorders pose a major global health risk and increase the medical burden, the discovery of novel inhibitors of pro-inflammatory factors through the evaluation of therapeutic agents is crucial.

Medicinal plants and their phytochemicals play a vital role in drug development for various diseases (Deeksha et al., 2022). The medicinal properties of many plants have been long recognized even though traditional users may not have understood the underlying mechanisms of action (Shakya, 2016). Modern pharmacology is increasingly drawn to ethnopharmacology due to its cost-effectiveness and reduced side effects. In fact, up to 50% of prescription drugs are derived from herbal sources (Verma et al., 2021). Traditional Indian medicine, particularly Ayurveda, has gained prominence for treating chronic illnesses, especially during the global SARS-CoV-2 pandemic, and has had a notable impact on global health (Deeksha et al., 2022). According to the WHO, 80% of the global population depends—directly or indirectly—on medicinal plants for primary healthcare, with approximately 21,000 plant species used medicinally (Deeksha et al., 2022; Verma et al., 2021). Bioassay-guided isolation is an efficient and effective strategy in drug discovery, allowing targeted isolation and identification of active components from complex plant extracts (Mani et al., 2022). This technique is particularly valuable when dealing with plant extracts containing diverse chemical constituents, enabling systematic fractionation and facilitating the investigation of their specific pharmacological activities (Atanasov et al., 2021).

*Zanthoxylum armatum* DC. is an aromatic, deciduous, spiny shrub that can grow up to 6 m tall. Commonly known as prickly ash or toothache tree, it is locally referred to as “Mukthrubi” in Manipuri (Singh et al., 2021). It belongs to the citrus family Rutaceae and holds both culinary and medicinal values. In Manipuri cuisine, its young shoots, leaves, flowers, and fruits are used—either fresh or cooked—in local delicacies, curries, fries, soups, meat and snail seasonings, and in a traditional salad called “Singju,” prized for its unique aroma and numbing, tingling sensation (Yumnam and Tipathi, 2012). In Sichuan cuisine, it enhances flavor and color, masks undesirable odors, serves as a preservative and health tonic, and stimulates appetite (Wei et al., 2020; Tian et al., 2013). Various parts of the plant—leaves, stems, fruits, and seeds—are widely used in traditional medicine to treat conditions such as cholera, dyspepsia, fever, cough, cold (Kala et al., 2005), indigestion, flatulence, depression (Zaidi et al., 2009), skin diseases, rheumatism (Baral and Kurmi, 2006), diarrhea, dysentery (Geweli and Awale, 2008), edema (Subedi, 2017), chest infections (Abbasi et al., 2010), tooth and gum problems (Sher et al., 2011), tick infestations, pneumonia (Sindhu et al., 2010), nerve and lung diseases (Aung et al., 2016), and as general tonics (Singh et al., 2016), owing to its deodorizing, sanitizing, and antiseptic properties. Several pharmacological studies have demonstrated the bioactivity of *Zanthoxylum armatum* DC., including antioxidant (Guleria et al., 2013; Sati et al., 2011; Batool et al., 2010), antibacterial (Irshad et al., 2021; Phuyal et al., 2020; Dhami et al., 2019; Nooreen et al., 2017; Srivastava et al., 2013), antifungal (Yasmeen et al., 2015; Prajapati et al., 2015; Barkatullah et al., 2012), anti-inflammatory (Sati et al., 2011), hepatoprotective (Talluri et al., 2019; Ranawat et al., 2010), and antiproliferative effects (Singh et al., 2022; Alam et al., 2017; Singh et al., 2015; Guo et al., 2011; Sati et al., 2011; Ramanujam and Ratha, 2008). Phytochemical analyses by various research workers have reported numerous bioactive compounds in the stem and bark of *Z. armatum* DC., including terpenoids such as α- and β-amyrins, β-amyrone, and lupeol (Tiwary et al., 2007; Kalia et al., 1999; Li et al., 1996); flavonoids such as 3,5-diactyltambulin and kaempferol (Li et al., 2006); alkaloids such as berberine, β- and γ-fagarine, chelerythrine, skimmianine, and zanthonitrile (Vashist et al., 2016; Ranawat et al., 2010; Li et al., 1996); lignans such as asarinin, sesamin, fargesin, L-asarinin, L-sesamin, and L-planinin (Bhatt et al., 2016; Vashist et al., 2016; Singh et al., 2013; Ranawat et al., 2010; Kalia et al., 1999; Li et al., 1996; Muller et al., 1996; Rao and Singh, 1994); coumarins such as xanthyletin, xanthoxyletin, alloxanthine, bergapten, and umbelliferone (Vashist et al., 2016; Li et al., 1996); sterols such as β-daucosterol, β-sitosterol, and β-sitosterol-β-D-glucoside (Vashist et al., 2016; Ranawat et al., 2010; Li et al., 1996); amides such as armatamide (Kalia et al., 1999); carbonyls such as cuminol and undecane-2-one (Kayat et al., 2016; Weyerstahl et al., 1999); and aromatics such as vanillic acid (Li et al., 1996). Given its wide range of bioactivities and diverse phytochemical composition, *Z. armatum* DC. holds promise as a priority medicinal plant for economic and social development, with the potential to support poverty reduction in rural communities. It is also necessary to validate traditional medicinal practices with modern scientific techniques to forge a solid connection between the traditional system of medicine and contemporary modern pharmacotherapy (Phuyal et al., 2019).

In this study, we aimed to identify the anti-inflammatory bioactive compound(s) and provide scientific evidence supporting the traditional use of *Z. armatum* DC. against inflammation. The plant was collected from the wild foothills of Nongmaiching Hill, Imphal East district, Manipur. A bioassay-guided phytochemical investigation of the stem led to the isolation of two lignans—sesamin and fargesin. Their anti-inflammatory activity was evaluated *in vitro* using conventional type 1 dendritic cells (cDC1) as no prior reports exist on this specific immune cell subtype. Despite the extensive ethnomedicinal usage and diverse pharmacological activities of *Z. armatum* DC., including anti-inflammatory properties, no systematic bioassay-guided studies have identified the specific anti-inflammatory compounds from the stem, nor have they explored their effects on conventional type 1 dendritic cells (cDC1)—a highly specialized and functionally critical subset of immune cells involved in the regulation of inflammation and antigen presentation. cDC1 cells play a central role in immune homeostasis and disease pathogenesis yet remain underexplored in plant-based anti-inflammatory research. To the best of our knowledge, this is the first study to isolate and evaluate specific lignans—namely, sesamin and fargesin—from the stem of *Z. armatum* using a bioassay-guided fractionation strategy, and to investigate their *in vitro* effects on cDC1 cells. This approach not only identifies the active principles behind the traditional use of this plant but also extends the scope of plant-based immunopharmacology into relatively uncharted cellular models. Therefore, by integrating ethnopharmacological knowledge with modern bioassay-guided isolation and immunological evaluation, in this study, we provide a novel and significant contribution to both natural product drug discovery and dendritic cell-targeted inflammation research.

## 2 Materials and methods

### 2.1 Collection and authentication of the plant sample

**FIGURE 1:**
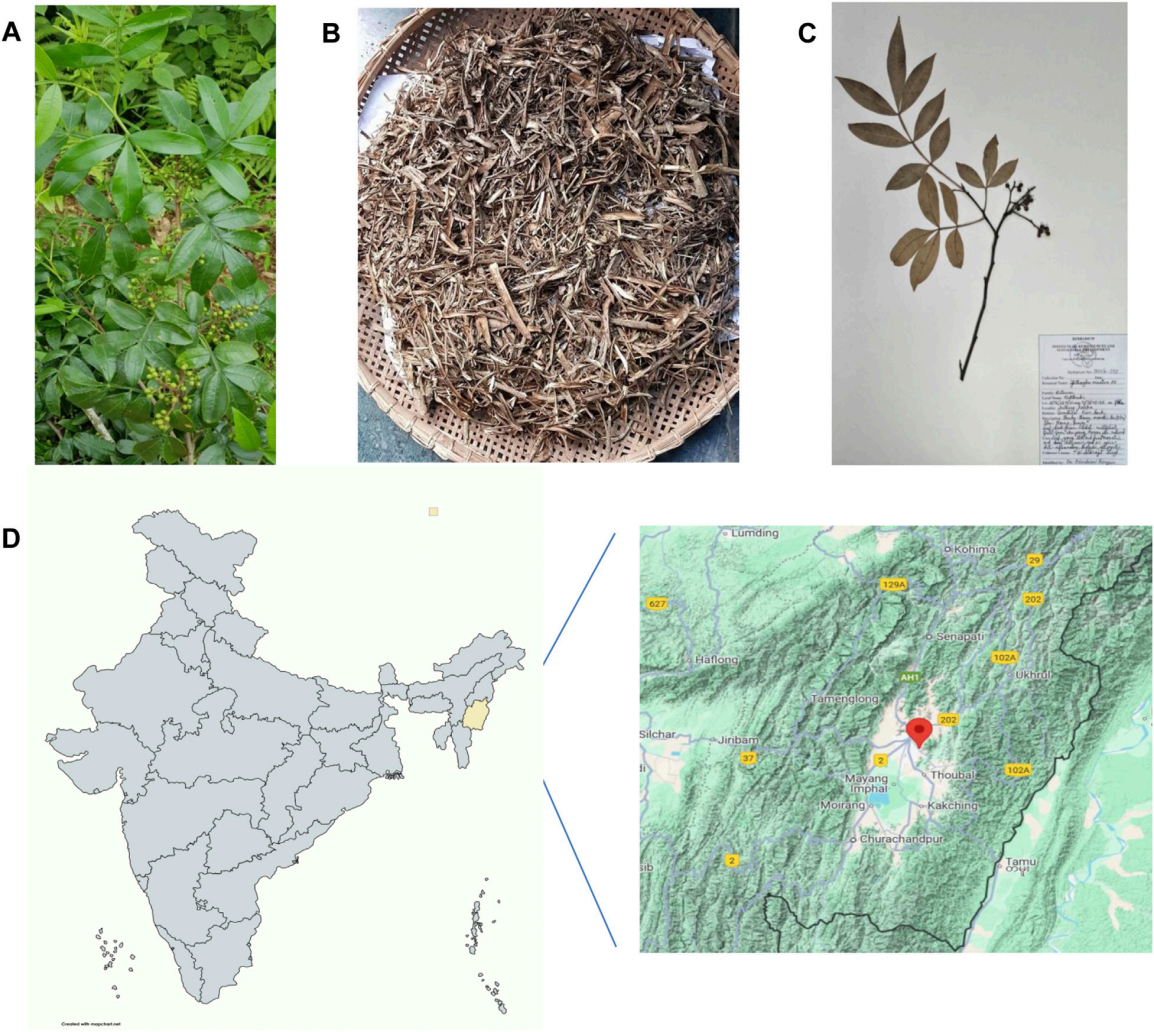
Collection, authentication, and processing of *Zanthoxylum armatum* DC. **(A)** Whole plant. **(B)** Shade-dried stem. **(C)** A specimen submitted to the herbarium at Plant Systematics and Conservation Laboratory, Plant Bioresources Division, Institute of Bioresources and Sustainable Development (IBSD), Imphal. **(D)** Geographical location of the sample collection site (24°46′08.9″N and 93°58′45.1″E), Imphal East, Manipur, India.

The *Zanthoxylum armatum* DC. plant specimen was collected in August 2018 from the Nongmaiching foothills, Imphal East, Manipur, India (latitude 24°46′08.9″N and longitude 93°58′45.1″E). August was chosen as the collection period because young branches reach optimal maturity—neither too soft and green nor too hard and woody. The taxonomic identity was confirmed using the World Flora Online database (www.worldfloraonline.org) and independently validated by the plant taxonomist, Dr. Biseshwori Thongam at Plant Systematics and Conservation Laboratory, Plant Bioresources Division, Institute of Bioresources and Sustainable Development (IBSD), Imphal. A voucher specimen was deposited in the herbarium at Plant Systematics and Conservation Laboratory, IBSD, Imphal (voucher number: IBSD/M-273) (Figure 1) (Supplementary Material).

### 2.2 Preparation of the hydroalcoholic stem extract

Stems were washed properly with tap water, shade-dried at room temperature for 15 days, and then ground into powder. Approximately 1.52 kg of the stem powder was macerated with 6 L of 70% methanol for 6 days at room temperature with constant shaking (Mani et al., 2022). The macerate was filtered through a Whatman filter paper, and the filtrate was concentrated under reduced pressure using a rotary evaporator (Buchi Rotavapor, Switzerland) to dryness. The final yield was 85 g of the crude extract from the 1.52 kg starting material, giving a percentage yield of approximately 5.59% (w/w). This crude hydromethanolic stem extract of *Z. armatum* DC. was assigned as “ZS.” The ZS crude extract was then subjected to primary fractionation using the liquid–liquid partition method with a gradient of organic solvents of increasing polarity, enabling the grouping of potential bioactive compounds for further column chromatography (Mani et al., 2022). From 80 g of the crude ZS extract, the yields were as follows: 13 g of the hexane fraction (ZSH, 16.25%), 35 g of the ethyl acetate fraction (ZSE, 43.75%), 15 g of the butanol fraction (ZSB, 18.75%), and 12 g of the methanol fraction (ZSM, 15%).

### 2.3 Phytochemical screening

The extract was screened for different plant phytochemicals using standard procedures (Shaikh and Patil, 2020; Banu and Cathrine, 2015). For each test, the extract was tested in three replicates, and the presence or absence of each class of compounds was recorded. Phytochemicals tested included alkaloids (Dragendorff’s test and Hager’s test), flavonoids (Shinoda test and alkaline test), phenolics (ferric chloride test and lead acetate test), carbohydrates (Molisch’s test), reducing sugars (Fehling’s test), non-reducing sugars (iodine solution test), proteins and amino acids (biuret and ninhydrin test), tannins (ferric chloride), steroids (Salkowski’s test), terpenoids (concentrated sulfuric acid and copper acetate test), glycosides (Keller–Kelliani test and 10% ammonia test), and saponin (foam test) (Supplementary Material).

### 2.4 Estimation of the total phenolic content (TPC)

The TPC of the hydromethanolic stem extract and its four fractions were estimated using the Folin–Ciocalteu method (Benabdallah et al., 2016). In brief, to 100 µL of the test sample, 500 µL of 10% solution of the Folin–Ciocalteu reagent (v/v) and 400 µL of 7.5% Na_2_CO_3_ (w/v) were mixed. After 30 min of incubation at room temperature, absorbance was measured at 765 nm. Each sample was analyzed in triplicate. Total phenolic contents were estimated from a gallic acid standard curve and expressed as µg gallic acid equivalents (GAE)/mL.

### 2.5 Estimation of the total flavonoid content (TFC)

Similarly, total flavonoid contents of the hydromethanolic stem extract and its four fractions were estimated using the aluminum chloride colorimetric method (Nurcholis et al., 2021). In brief, 1.5 mL of methanol, 0.5 mL of the sample, and 2.8 mL of distilled water were mixed. To this mixture, 0.1 mL of 10% aluminum chloride and 0.1 mL of 1M potassium acetate were added. After 30 min of incubation at room temperature, absorbance was observed at 415 nm. Each sample was tested in triplicate. Total flavonoid contents were estimated from a standard calibration curve of quercetin (10–100 μg/mL) and expressed as µg quercetin equivalents (QE)/mL.

In the preliminary phytochemical analysis, the plant extract ZS was found to be rich in phenolics and flavonoids, which are key secondary metabolites of plants and considered natural anti-inflammatory agents. Among the four fractions, the ethyl acetate fraction (ZSE) showed the highest phenolic content (100 ± 1.14 µg gallic acid equivalent) and the flavonoid content (101.33 ± 0.50 µg quercetin equivalent). Therefore, both ZS and ZSE were further subjected to GCMS-based phytochemical fingerprinting.

### 2.6 Gas chromatography–mass spectrometry-based phytochemical fingerprinting

For gas chromatography–mass spectrometry (GC–MS)-based phytochemical profiling, 2 mL each of 20 mg/mL stock solutions of the hydromethanolic stem extract (ZS) and its ethyl acetate fraction (ZSE) were subjected to the GC–MS analysis (Shimadzu GCMS QP-2010 Plus with Thermal Desorption System TD 20, Auto-injector System AOC-2oi, and Auto-sampler Unit AOC-2os) at the Advanced Instrumentation Research Facility (AIRF), Jawaharlal Nehru University, New Delhi.

The column used was Rtx-5 MS (Restek Corporation), with a diameter of 0.25 mm, a length of 30 m, and a film thickness of 0.25 µm. The oven temperature was increased to 280 °C from initial 50 °C at the rate of 5 °C/min, and the pressure was maintained at 69 KPa. The temperature of the detector interface was 270 °C, and helium was used as the carrier gas at a purge flow of 3 mL/min. The temperature of injection was 260 °C, and the auto-sampler unit used 1 µL of the sample at a split ratio of 1:10, with a flow rate of 1.21 mL/min.

For mass spectrometry, the temperature was ramped from 50 °C (hold for 2 min) to 210 °C at 3 °C/min and then up to 280 °C at 8 °C/min, with a final hold of 8 min. The temperature of the ion source was 230 °C and that of the interface was 270 °C, with a cut time of 2.50 min and a threshold of 1000. The total length of the run was 74 min, with a scan speed of 3,333 amu/sec and a scan range of 40–650 m/z.

The Shimadzu GCMS QP-2010 Plus acquired the chromatograms and mass spectra, which were processed using GCMS Solutions post-run analysis software. The identification of compounds was based on their relative retention indices of n-alkanes and by comparison with mass-spectral libraries (NIST14, FFNSC2), using a 75% similarity index cutoff (Chikowe et al., 2024) (Supplementary Table S1).

### 2.7 Bioassay-guided isolation of active components

#### 2.7.1 Albumin denaturation inhibition assay

The heat-induced albumin denaturation inhibition ability of the extract (ZS) and its four fractions (ZSH, ZSE, ZSB, and ZSM) was evaluated following the method described by Kola et al. (2022) with slight modifications. In brief, 500 µL of 1% aqueous bovine serum albumin (Sigma) was mixed with 500 µL of the test sample. The pH was adjusted to 6.8 using 1N HCl. The mixture was incubated for 20 min at 37 °C and then for 20 min at 57 °C, followed by cooling. Absorbance was observed at 660 nm. Water and diclofenac were taken as the control and standard references, respectively. Both standard and samples were tested at different concentrations (10–100 μg/mL) in triplicates.

#### 2.7.2 Heat-induced hemolysis inhibition assay

The heat-induced hemolysis inhibition assay was also conducted with the extract and its four fractions following the method by Righi et al. (2021), with minor modifications. In brief, 500 µL of 10% red blood cell (RBC) suspension and 500 µL of the test sample were mixed and incubated at 56 °C for 30 min. After cooling, the mixture was centrifuged at 2,500 rpm for 5 min, and the absorbance of the supernatant was observed at 560 nm. Normal saline and diclofenac were taken as the control and standard references, respectively. For both standard and tested samples, each concentration (10–100 μg/mL) was tested in triplicate.

The percentage inhibition was calculated using the following formula:

$$\frac{\%\;inhibition=Control\;Absorbance-Test\;Absorbance\;\text{×}\;100}{Control\;Absorbance}$$

The IC50 values were calculated by plotting a linear graph of % inhibition against the concentration of the sample.

#### 2.7.3 Column chromatography

Among the four fractions, the ethyl acetate fraction (ZSE) showed significant bioactivity and was therefore subjected to 60–120 mesh silica gel column chromatography (Borosilicate glass column from Sigma), with a gradient of hexane:ethyl acetate in the following ratios: 10:0, 9.5:0.5, 9:1, 8.5:1.5, 8:2, 7.5:2.5, 7:3, 6.5:3.5, 6:4, 5.5:4.5, 5:5, 4.5:5.5, 4:6, 3.5:6.5, 3:7, 2.5:7.5, 2:8, 1.5:8.5, 1:9, 0.5:9.5, and 0:10. This process yielded 21 subfractions and were labeled ZSE1–ZSE21.

**FIGURE 2:**
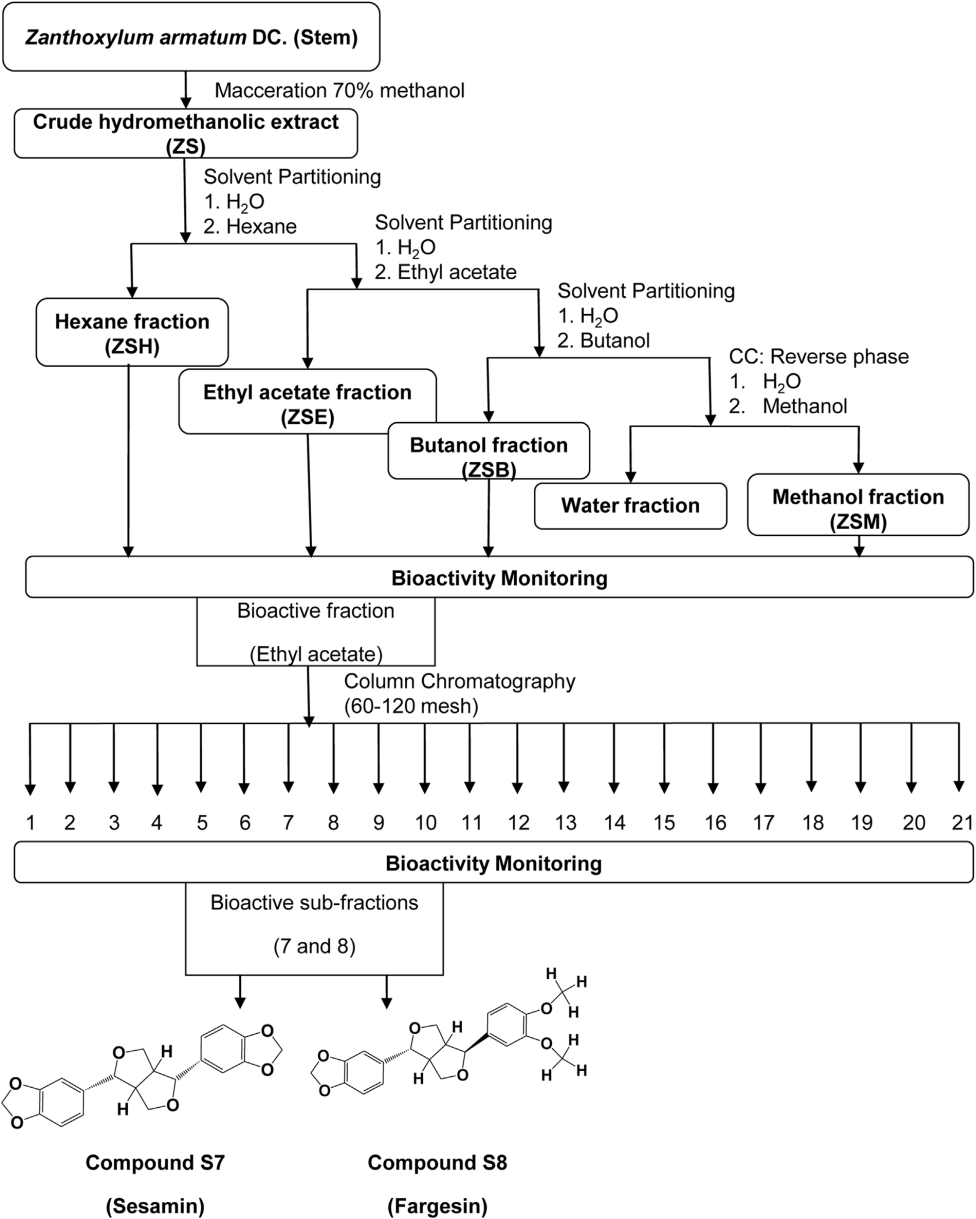
Flowchart of bioassay-guided isolation of two active compounds, S7 and S8, from the hydromethanolic stem extract of *Zanthoxylum armatum* DC.

Subfractions ZSE7 and ZSE8 showed significant activity and were further subjected to a second round of column chromatography using 100–200 mesh silica. Subfraction ZSE7 yielded a crystalline component with significant activity and was assigned as S7. Similarly, sub-fraction ZSE8 yielded another active crystalline component and was assigned as S8. A detailed workflow is presented in Figure 2.

### 2.8 HPLC purification

Purification of isolated components S7 and S8 was performed using a high-performance liquid chromatography (HPLC) system (SHIMADZU LC-20AP 02027/02028 pump with SPD-20A40766 UV–VIS detector) at the Central Instrumentation Facility, IBSD, Imphal. The mobile phase consisted of 80% methanol and 20% water (pH 7). The flow rate was 2 mL/min, and the column used was SHIMADZU C-18, 5 μm, 10 × 250 mm maintained at 40^0^C. Elutes of the major peaks were pooled and concentrated using a rotary vacuum evaporator followed by lyophilization (Labconco) to remove the residual solvent (Supplementary Table S2).

### 2.9 Single crystal x-ray diffractometry (XRD)

Recrystallization of purified compounds S7 and S8 was performed using the solvent layering method with ethyl acetate and dichloromethane (1:1). Suitable single crystals of both S7 and S8 were analyzed using an Enraf (Bruker) Nonius CAD4-MV31 XRD system coupled with an FR 590 generator, a goniometer, an interface of CAD4F, and a printer- and plotter-equipped microVAX3100 available at Sophisticated Analytical Instrument Facility (SAIF), IIT Madras. The scintillation counter was used as the detector, a single crystal was mounted on a goniometer head’s fixed thin glass fiber, and cell dimensions and orientations were determined using 25 reflections. An IBM compatible PC/AT 486 attached with MicroVAX facilitated data processing. Mercury 4.2.0 software was used for structural visualization and analysis of the procured data, whereas ORTEP-3 was used to draw the molecular structures.

### 2.10 Nuclear magnetic resonance spectrometry (NMR)

Solution-state 1D and 2D nuclear magnetic resonance (NMR) spectra of both compounds (S7 and S8) were analyzed using a Bruker Advance III 500 MHz spectrometer, coupled with an 11.7 T actively shielded superconducting magnet (5.4-cm standard bore), built-in cryo-shims, and a 4-channel RF console with stable auto-gradient shimming deuterium lock capable of triple resonance. A 5-mm quadrupole inverse (QXI) probe with gradient capability was used. The solvent used was deuterated DMSO, and then, the acquired spectra were processed using TOPSPIN 2.1 Windows XP-based software at Sophisticated Analytical Instrument Facility (SAIF), IIT Madras. TOPSPIN 4.3.0 software was used for viewing and analyzing the procured data; compounds were identified by comparing the spectra with those reported in the previously published literature, and ChemDraw Ultra 8.0 was used to draw the molecular structures.

### 2.11 Mass spectrometry

Mass spectrometry was carried out using a Waters LC-QTOF-HRMS system coupled with an ACQUITY H-CLASS PLUS UPLC and a XevoG2 XS QTOF mass spectrometer at the Sophisticated Analytical Instrument Facility (SAIF), IIT Madras. The UPLC system comprised a binary solvent manager, a sample and column manager, and a PDA detector, whereas the mass spectrometer featured StepWave ion optics, an XS collision cell, and QuanToF technology, providing a mass range of 20 to 1,00,000 m/z, an acquisition rate of 30 spectra per second, and a mass accuracy of 1 ppm.

### 2.12 *In vitro* anti-inflammatory mechanism study

#### 2.12.1 Cell culture

The murine cDC1 cell line MuTuDC1940 (thereafter referred to as MuTuDCs) used in this study was a gift from Prof. Hans Acha-Orbea. The cell culture medium was prepared by mixing incomplete Iscove’s modified Dulbecco’s medium (IMDM) with fetal bovine serum (FBS), HEPES, penicillin–streptomycin (PS), sodium bicarbonate, and β-mercaptoethanol. The cell line was kept at 37 °C in a humidified incubator with 5% CO_2_. The cells were dissociated with a short incubation period of 3 min in a nonenzymatic manner. Following a 5-min centrifugation at 2500 *g*, cells were resuspended in fresh complete IMDM and cultured in 100-mm culture dishes. All experiments were conducted in three biological replicates (n = 3 independent experiments).

#### 2.12.2 Cell viability

Stock solutions of 100 mg/mL of both compounds were prepared by dissolving 10 mg of each of compound in 100 μL DMSO. A sub-stock of 1 mg/mL for each compound was then prepared using incomplete IMEM to finally obtain a working concentration of 25 μg/mL. Experimental groups were organized as follows: an unstimulated group (uns), comprising dendritic cells and complete IMDM; a stimulated group (CpG), comprising CpG (TLR9 agonist)-stimulated dendritic cells and complete IMDM; medication groups (S7 and S8), consisting of CpG-stimulated MutuDCs treated with 100 μL medium containing 25 μg/mL of each of the compounds.

MutuDCs were seeded at a density of 1 × 10^5^ cells/well in 96-well plates. After overnight incubation, cells were washed with PBS, treated with 25 μg/mL of the compounds, followed by the addition of CpG-B (1 μg/mL), and incubated at 37 °C for another 6 h (*n* = 3). As the MutuDC is GFP+, the average intensity of GFP signals was evaluated using flow cytometry, as a proxy for cell viability. Each condition was tested in three biological replicates (n = 3).

#### 2.12.3 Flow cytometry assay

The anti-inflammatory effects of sesamin and fargesin were evaluated in MutuDC stimulated with CpG-B. Cells (1 × 10^5^/well) were pre-cultured overnight; the next morning, the cells were treated with the bioactive compounds at a concentration of 25 μg/mL for 1 h. Following treatment, cells were exposed to CpG-B stimulation (or no stimulation for control conditions) for an additional 6 h. After dissociating the cells from plates, those were washed with FACS buffer (3% FBS in PBS), followed by the addition of the appropriate flow cytometry antibody specific for CD80 (a cell surface marker) to approximately 50 μL of cell suspension, and incubated at 4 °C for 30 min. For intracellular staining of IL12p40/70, the cells were first fixed with 2% paraformaldehyde, followed by permeabilization using 1X permeabilization buffer. The fixed cells were then stained with fluorochrome-tagged antibody specific for IL12p40/70. The cells were incubated with antibodies for 30 min in dark at room temperature for optimal staining. After the incubation time was over, the cells were washed twice with the FACS buffer. After staining, the cells were analyzed within 24 h. All flow cytometry analyses were performed in triplicates (n = 3) using the Cytek Aurora, and the acquired data were analyzed using FlowJo-X software.

### 2.13 *In silico* physiochemical, pharmacokinetic, pharmacodynamic, toxicity, and drug-likeness prediction study

ADMETlab 2.0 was used for the prediction of absorption, distribution, metabolism, excretion, and toxicity—simply the ADMET profile of the two isolated phyto-compounds, S7 and S8, and drug-likeness was determined using the Lipinski Rule-of-5. Boiled-egg plot prediction for the isolated compounds sesamin and fargesin was also performed using the swissADME server. Compounds in agreement with such standard parameters have been reported to show better bioavailability and pharmacokinetics (Ibrahim et al., 2021). Physiochemical characteristics of the compounds, such as molecular weight, hydrogen bond acceptor, hydrogen bond donor, rotatable bonds, and topological polar surface area, are also important for determining oral absorption. Absorption parameters also included Caco-2 and MDCK cell permeability, P-glycoprotein substrate and inhibitor, and human intestinal absorption. Distribution parameters included blood–brain barrier penetration, plasma protein binding, volume distribution value, and fraction bound in plasma. Metabolism parameters included five isozymes, namely, 1A2, 3A4, 2C9, 2C19, and 2D6, which belong to the cytochrome P450 family. Excretion parameters included clearance and half-life, whereas toxicity parameters included hepatotoxicity, drug-induced liver damage, Ames toxicity, rat oral toxicity, skin sensitization, carcinogenicity, eye corrosion, and respiratory toxicity. Parameters of drug-likeness are quantitative estimate of drug-likeness, synthetic accessibility, and fraction of sp3 carbon atoms.

## 3 Statistical analysis

Statistical analyses were performed using GraphPad Prism v8.0.2. and MS Excel 2007. Each experiment was conducted in triplicate (n = 3). All data are presented as mean ± standard deviation (SD). Normality of data distribution was assessed using the Shapiro–Wilk test. For comparisons between groups, one-way ANOVA followed by Tukey’s *post hoc* test (for equal variances) or Tamhane’s T2 test (for unequal variances) was used. Homogeneity of variances was checked using Levene’s test. A p-value of <0.05 was considered statistically significant. Outlier analysis was conducted using Grubbs’ test in the GraphPad Outlier Calculator.

## 4 Results

### 4.1 Preliminary phytochemical profiling

Standard phytochemical tests for qualitative screening of the extract are presented in Supplementary Table S3. The hydromethanolic stem extract of *Z. armatum* DC. showed the presence of various phytoconstituents, such as phenolic compounds, flavonoids, alkaloids, terpenoids, and glycosides, which are responsible for its medicinal properties. The presence of carbohydrates, proteins, and anthraquinone glycosides was not detected, possibly due to their limited solubility in high polar solvent such as methanol.

### 4.2 Total phenolic and flavonoid contents

The total phenolic content and the total flavonoid content of the tested samples are represented in Table 1. The ethyl acetate fraction (ZSE) showed the highest phenolic content (100 ± 1.14 SD µg gallic acid equivalent) and the flavonoid content (101.33 ± 0.50 SD µg quercetin equivalent), followed by the hexane fraction (ZSH) (phenolic content 51 ± 0.90 SD µg gallic acid equivalent and flavonoid content 49.80 ± 0.78 SD µg quercetin equivalent), methanol fraction (ZSM) (phenolic content 50 ± 0.90 SD µg gallic acid equivalent and flavonoid content 46.19 ± 0.32 SD µg quercetin equivalent), and butanol fraction (ZSB) (phenolic content 43.91 ± 0.62 SD µg gallic acid equivalent and flavonoid content 45.49 ± 0.32 SD µg quercetin equivalent). The standard gallic acid curve and the quercetin curve are presented in Supplementary Figure S1.

**TABLE 1:** Quantification of the total phenolic content and the total flavonoid content of the hydromethanolic stem extract and its four fractions.

Sl. no.	Sample	Total phenolic content (µg/mL GAE ± SD)	Total flavonoid content (µg/mL QE ± SD)
1	ZS	59.25 ± 0.66	66.80 ± 0.73
2	ZSH	51 ± 0.90	49.80 ± 0.78
3	ZSE	100 ± 1.14	101.33 ± 0.50
4	ZSB	43.91 ± 0.62	45.49 ± 0.32
5	ZSM	50 ± 0.90	46.19 ± 0.32

### 4.3 Gas chromatography–mass spectrometry-based phytochemical profiling

**FIGURE 3:**
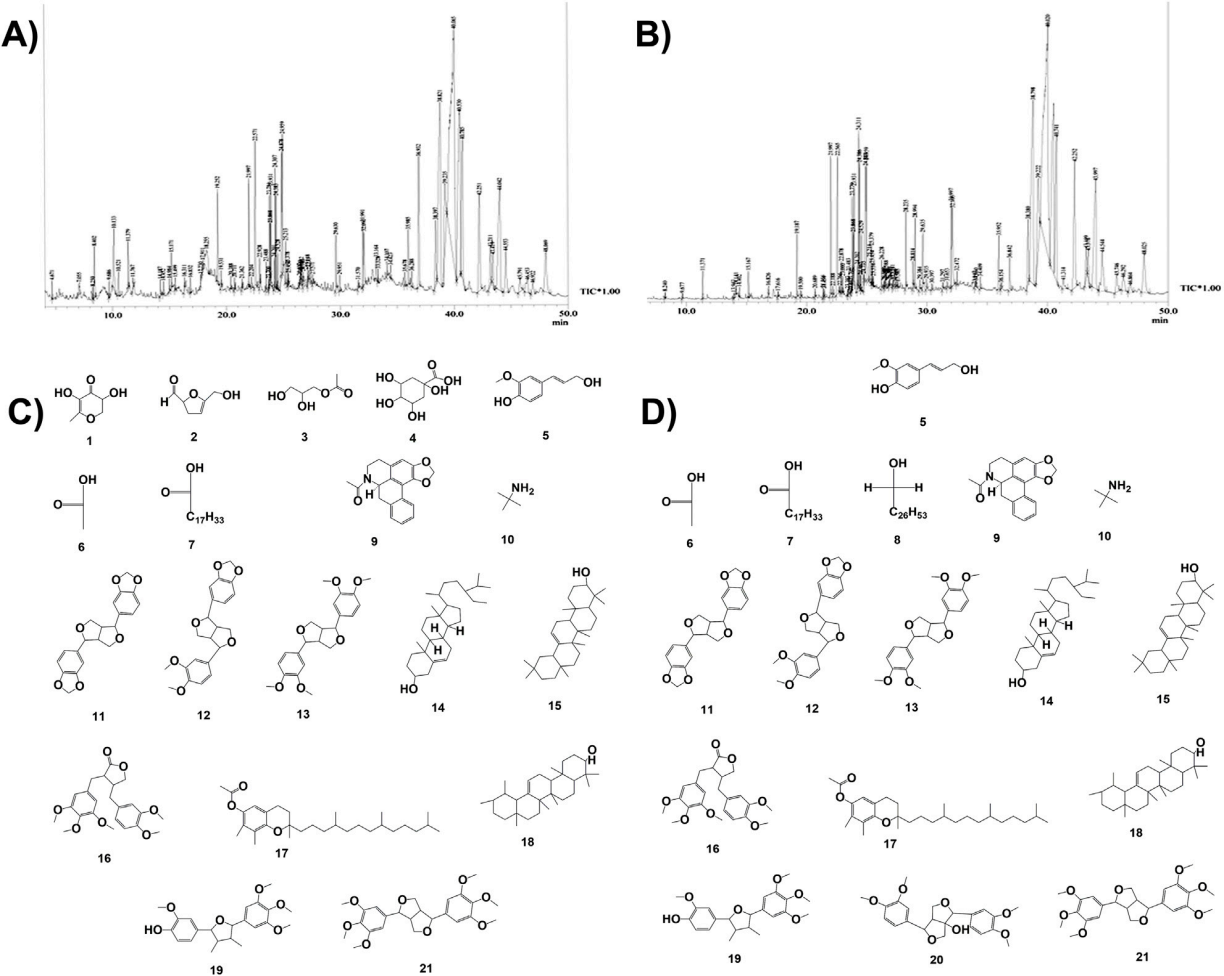
GCMS-based phytochemical fingerprinting. **(A)** Chromatogram of GC–MS for the hydromethanolic stem extract (ZS) of *Zanthoxylum armatum* DC. **(B)** Chromatogram of GC–MS for the ethyl acetate fraction (ZSE) of the hydromethanolic stem extract of *Zanthoxylum armatum* DC. **(C)** Some of the major compounds present in the hydromethanolic stem extract (ZS) of *Zanthoxylum armatum* DC. **(D)** Some of the major compounds present in the hydromethanolic stem extract (ZS) of *Zanthoxylum armatum* DC. and its ethyl acetate fraction (ZSE).

GC–MS chromatograms of both the hydromethanolic extract and its ethyl acetate fraction are represented in Figure 3. The GC–MS analysis of the hydromethanolic stem extract and its ethyl acetate fraction detected 19 and 17 major compounds, respectively. The 15 major compounds, namely, **(E)**-conipheryl alcohol, hexadecanoic acid, octadecanoic acid, n-acetylanonaine, 2-propanamine, 2,6-bis(3,4-methylenedioxyphenyl)-3,7-dioxybicyclo[3.3.0]octane, fargesin, 1H,3H-furo[3,4-c]furan, 1,4-bis(3,4-dimethoxyphenyl)tetrahedro-[1R-(1.alpha.,3a.beta.,4.alpha.,6a.alpha.)], gamma.-sitosterol, alpha.- and beta.-amyrin, (3R,4R)-4-(3,4-dimethoxybenzyl)-3-(3,4,5-trimethoxybenzyl)dihydrofuran-2(3H)-one, gamma.-tocopherol, 4-((2R,3R,4R,5R)-3,4-dimethyl-5-(3,4,5-trimethoxyphenyl)tetrahydrofuran-2-yl)-2-methoxyphenol, and yangambin, are common to both the hydromethanolic extract and its ethyl acetate fraction. A furo-furan lignan, that is, fargesin, has the highest concentration in both the hydromethanolic stem extract and its ethyl acetate fraction, with percentage areas of 26.49% in the hydromethanolic extract and 27.88% in the ethyl acetate fraction. Fargesin is followed by another furo-furan lignan, 1H,3H-furo[3,4-c]furan,1,4-bis(3,4-dimethoxyphenyl)tetrahedro-,[1R-(1.alpha.,3a.beta.,4.alpha.,6a.alpha.)] and 2,6-bis(3,4-methylenedioxyphenyl)-3,7-dioxybicyclo(3.3.0)octane with percentage areas of 13.87% and 11.28% in the stem extract and 14.42% and 11.38% in the ethyl acetate fraction, respectively. Lignans constituted 55.79% and 58.22% of the total percent area concentration in the extract and the ethyl acetate fraction, respectively (Supplementary Table S4).

### 4.4 Bioassay-guided isolation of active components

#### 4.4.1 RBC membrane lysis inhibition test

**FIGURE 4:**
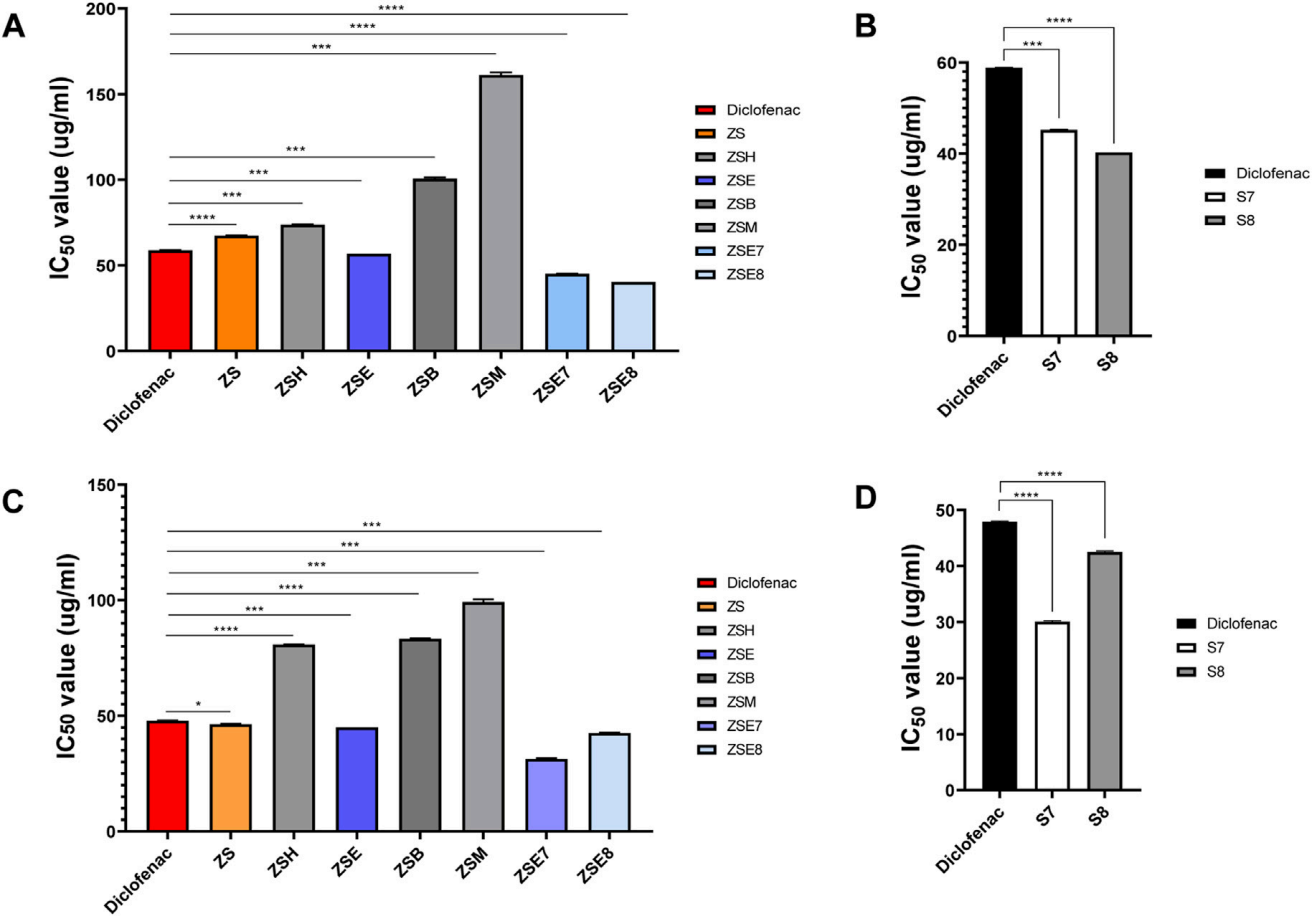
Bioactivity monitoring. **(A)** Heat-induced hemolysis inhibition activity of the hydromethanolic stem extract, its four fractions, and two active subfractions from the stem of *Zanthoxylum armatum* DC. **(B)** Heat-induced hemolysis inhibition activity of two isolated phyto-components S7 = sesamin and S8 = fargesin. **(C)** Heat-induced albumin denaturation inhibition activity of the hydromethanolic stem extract, its four fractions, and two active subfractions from the stem of *Zanthoxylum armatum* DC. **(D)** Heat-induced albumin denaturation inhibition activity of two isolated phyto-components S7 = sesamin and S8 = fargesin. ZS, *Zanthoxylum armatum* DC. hydromethanolic crude extracts of the stem; ZSH, hexane fraction of ZS; ZSE, ethyl acetate fraction of ZS; ZSB, butanol fraction of ZS; ZSM, methanol fraction of ZS. The standard anti-inflammatory drug diclofenac was used as the positive control. Each value expresses the mean ± SD of triplicates.

The *in vitro* anti-inflammatory potential of the hydromethanolic stem extract, its four fractions, two active subfractions of the ethyl acetate fraction, and two isolated compounds is shown in Table 2; Figure 4. The standard anti-inflammatory drug diclofenac showed an active RBC membrane lysis inhibition potential, with IC50 of 58.86 ± 0.11 SD µg/mL. Among the four fractions, the ethyl acetate fraction (ZSE) showed the highest anti-inflammatory potential, with IC50 of 56.67 ± 0.00 SD µg/mL. Column chromatography of the ethyl acetate fraction yielded 21 subfractions. Among them, the subfractions ZSE7 and ZSE8 showed strong anti-inflammatory activity, with IC50 of 45.09 ± 0.17 SD µg/mL and 40.24 ± 0.01 SD µg/mL, respectively, in the RBC membrane lysis inhibition test. Furthermore, column chromatography yielded two active compounds, S7 and S8. Both showed strong anti-inflammatory potentials compared to that of the standard anti-inflammatory drug, diclofenac. S7 showed IC50 of 45.19 ± 0.29 SD µg/mL, and S8 showed IC50 of 40.22 ± 0.00 SD µg/mL.

**TABLE 2:** Percentage inhibition of RBC membrane lysis by the crude hydromethanolic stem extract, its fractions, active subfractions, and isolated active phyto-components from the stem of *Zanthoxylum armatum* DC.

Parameter (µg/mL)	Percentage inhibition of RBC membrane lysis									
	Diclofenac	ZS	ZSH	ZSE	ZSB	ZSM	SE7	SE8	S7	S8
100	67.46 ± 0.41	57.17 ± 0.41	25.59 ± 0.41	55.74 ± 0.41	48.56 ± 1.09	29.42 ± 0.41	65.78 ± 0.82	66.02 ± 0.41	65.31 ± 0.41	70.09 ± 1.09
80	62.91 ± 0.41	54.54 ± 0.82	20.09 ± 0.82	52.63 ± 0.71	44.73 ± 0	17.22 ± 0.41	62.91 ± 0.82	63.39 ± 0.71	63.15 ± 0.41	63.15 ± 0.41
60	51.43 ± 0.41	47.36 ± 0.41	19.13 ± 1.09	50.95 ± 0.41	35.40 ± 0.71	13.39 ± 1.09	61.72 ± 1.09	60.76 ± 1.09	62.43 ± 0.41	62.20 ± 0.41
50	38.99 ± 1.89	38.03 ± 0.41	16.74 ± 0.71	48.08 ± 0.82	33.96 ± 0.71	−0.24 ± 0.82	55.97 ± 0.41	59.56 ± 0.41	55.97 ± 0.41	60.28 ± 0.41
40	27.51 ± 0.71	27.510 ± 0.71	14.11 ± 0.41	38.99 ± 0.71	23.20 ± 0.71	5.73 ± 0.82	43.77 ± 0.41	49.75 ± 0.15	43.53 ± 0.414	49.75 ± 0.17
20	22.48 ± 0.71	25.35 ± 1.43	−93.78 ± 1.43	31.57 ± 0.41	23.44 ± 0.41	7.65 ± 0.41	33.96 ± 0.71	38.51 ± 0.41	33.96 ± 0.71	38.51 ± 0.41
10	20.81 ± 1.09	23.44 ± 1.09	−169.14 ± 3.58	29.18 ± 0.41	21.28 ± 0.41	0.95 ± 1.36	28.94 ± 0.00	31.57 ± 0.41	28.94 ± 0	31.57 ± 0.41
IC50	58.86 ± 0.11	67.34 ± 0.10	73.76 ± 0.40	56.67 ± 0.00	100.63 ± 1.37	161.20 ± 2.59	45.09 ± 0.17	40.24 ± 0.01	45.19 ± 0.29	40.22 ± 0.00

All values are mean ± SD (n = 3); ZS, *Zanthoxylum armatum* DC. hydromethanolic crude extracts of stem; ZSH, hexane fraction of ZS; ZSE, ethyl acetate fraction of ZS; ZSB, butanol fraction of ZS; ZSM, methanol fraction of ZS; SE7, active subfraction number 7 from ZSE; SE8, active subfraction number 8 from ZSE; isolated compound S7 = sesamin; isolated compound S8 = fargesin. Standard anti-inflammatory drug diclofenac was used as the positive control.

#### 4.4.2 Albumin anti-denaturation test

The standard anti-inflammatory drug (diclofenac) showed an active albumin denaturation inhibition potential, with IC50 of 47.90 ± 0.15 SD µg/mL. Similarly, the ethyl acetate fraction (ZSE) showed the highest albumin denaturation inhibition potential, with IC50 of 45.00 ± 0.00 SD µg/mL. Active sub fractions ZSE7 and ZSE8 showed strong albumin denaturation inhibition activity, with 31.38 ± 0.55 SD µg/mL and 42.60 ± 0.14 SD µg/mL, respectively. The isolated compounds (S7 and S8) also showed strong anti-inflammatory potentials compared to that of the standard anti-inflammatory drug (diclofenac). S7 showed IC50 of 30.08 ± 0.15 SD µg/mL, and S8 showed IC50 of 42.50 ± 0.19 SD µg/mL, indicating that the isolated phyto-components, S7 and S8, have high anti-inflammatory potentials compared to that of the standard drug, diclofenac (Table 3; Figure 4).

**TABLE 3:** Percentage inhibition of protein denaturation by the crude hydromethanolic stem extract, its fractions, active subfractions, and isolated active phyto-components from the stem of *Zanthoxylum armatum* DC.

Parameter (µg/mL)	Percentage inhibition of protein denaturation									
	Diclofenac	ZS	ZSH	ZSE	ZSB	ZSM	SE7	SE8	S7	S8
100	69.98 ± 0.27	67.93 ± 0.27	45.18 ± 0.54	64.29 ± 0.27	61.29 ± 0.54	48.81 ± 0.94	77.56 ± 0.27	77.56 ± 0.27	77.56 ± 0.27	78.83 ± 0.27
80	69.03 ± 0.54	63.82 ± 0.27	43.28 ± 0.27	57.34 ± 0	47.70 ± 0.27	44.54 ± 1.64	76.46 ± 0.54	77.25 ± 0.47	76.61 ± 0.27	77.88 ± 0.27
60	61.13 ± 0.47	58.76 ± 0.47	−106.95 ± 2.73	52.13 ± 0.47	47.07 ± 0.72	45.97 ± 0.82	74.24 ± 0.27	74.40 ± 0.82	74.56 ± 0.54	74.88 ± 0.82
50	51.50 ± 0.27	53.39 ± 0.27	−77.72 ± 2.36	50.55 ± 0.27	44.39 ± 3.32	39.02 ± 3.15	73.14 ± 0.72	66.35 ± 0.47	73.93 ± 0.94	66.82 ± 0.94
40	44.39 ± 0.54	43.91 ± 0.27	27.96 ± 0.94	49.44 ± 0.27	41.70 ± 0.94	37.75 ± 0.54	57.03 ± 0.98	44.23 ± 0.27	59.08 ± 0.54	44.39 ± 0.27
20	41.54 ± 0.98	41.70 ± 0.94	−27.96 ± 0.94	48.97 ± 0.27	33.96 ± 0.72	39.02 ± 0.27	40.75 ± 0.47	26.22 ± 0.27	40.75 ± 0.47	27.64 ± 1.19
10	23.85 ± 0.98	33.96 ± 0.72	−77.72 ± 2.36	48.34 ± 0.47	−4.10 ± 0.54	34.59 ± 0	9.32 ± 0.72	−2.05 ± 1.97	11.37 ± 0.82	5.21 ± 0.47
IC50	47.90 ± 0.15	46.41 ± 0.28	80.88 ± 0.14	45.00 ± 0.00	83.36 ± 0.28	99.20 ± 2.10	31.38 ± 0.55	42.60 ± 0.14	30.08 ± 0.15	42.50 ± 0.19

All values are mean ± SD (n = 3); ZS, *Zanthoxylum armatum* DC. hydromethanolic crude extracts of stem; ZSH, hexane fraction of ZS; ZSE, ethyl acetate fraction of ZS; ZSB, butanol fraction of ZS; ZSM, methanol fraction of ZS; SE7, active subfraction number 7 from ZSE; SE8, active subfraction number 8 from ZSE; isolated compound S7 = sesamin; isolated compound S8 = fargesin. Standard anti-inflammatory drug diclofenac was used as the positive control.

### 4.5 Structure elucidation of active compounds

**FIGURE 5:**
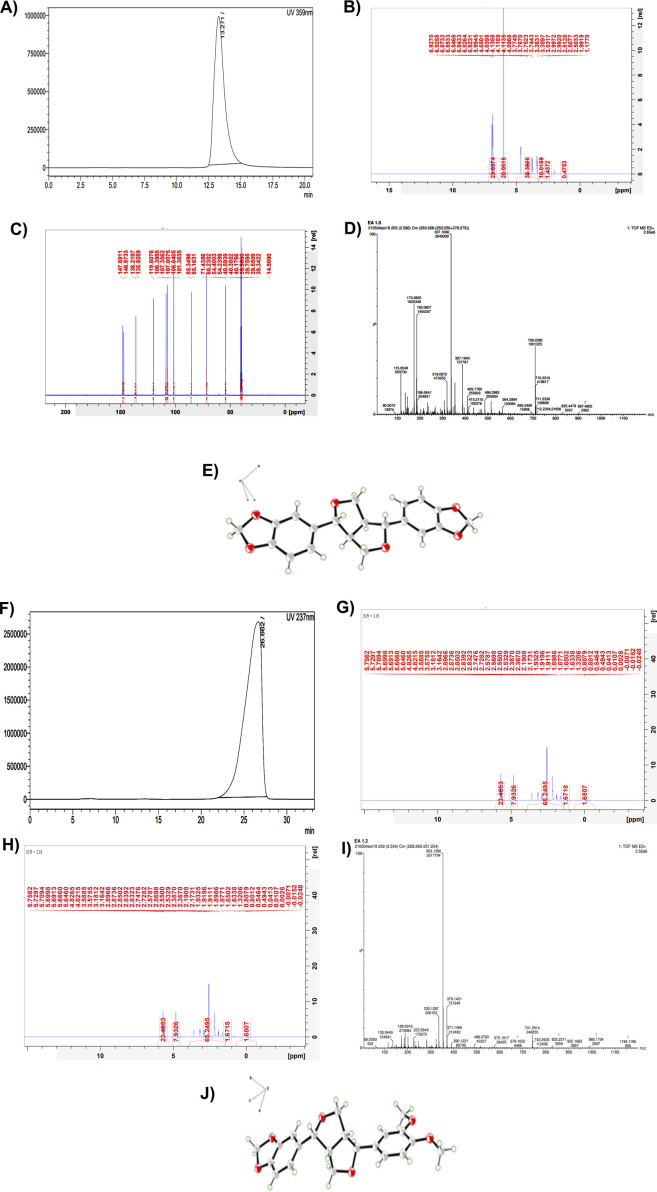
Part 1: Structure elucidation of the compound S7 isolated from the ethyl acetate fraction of the hydromethanolic stem extract of *Zanthoxylum armatum* DC. **(A)** HPLC chromatogram of S7. **(B)** H-NMR of S7. **(C)** C-NMR of S7. **(D)** Mass spectra of S7. **(E)** ORTEP diagram drawn with 50% thermal ellipsoid of isolated compound S7 = sesamin. Part 2: Structure elucidation of the compound S8 isolated from the ethyl acetate fraction of the hydromethanolic stem extract of *Zanthoxylum armatum* DC. **(F)** HPLC chromatogram of S8. **(G)** H-NMR of S8. **(H)** C-NMR of S8. **(I)** Mass spectra of S8. **(J)** ORTEP diagram drawn with 50% thermal ellipsoid of isolated compound S8 = fargesin.

Single crystal X-ray crystallography data, nuclear magnetic resonance spectra, and mass spectra of S7 and S8 were used for structure elucidation. Crystallography data from the isolated compounds are provided in Table 4, through structural analysis using X-ray crystallography; the ORTEP diagrams are also drawn with 50% thermal ellipsoid in Figure 5 (Supplementary Figures S2–S9).

**TABLE 4:** Crystallographic data on the isolated compounds from the ethyl acetate fraction of the hydromethanolic stem extract of *Zanthoxylum armatum* DC.

Parameter	S7	S8
Formula	C_20_H_18_O_6_	C_21_H_22_O_6_
Weight	354.34	370.38
Crystal system	Monoclinic	Orthorhombic
Space group	P21	P21 21 21
a (A^°^)	10.0701 (11)	5.9609 (3)
b (Å)	6.9290 (7)	9.3515 (6)
c (Å)	11.8864 (11)	32.763 (2)
α (°)	90	90
β (°)	93.489 (4)	90
γ (°)	90	90
V (Å3)	827.84 (15)	1826.33 (19)
Z	2	4
Density (Mgm^-3^)	1.422	1.347
Absorption coefficient (mm^-1^)	0.105	0.099
F (0000)	372	784
Total number of reflections	3396	3204
Reflections, I > 2*σ* (1)	2788	2210
Maximum 2*θ* (°)	26.383	25.027
Ranges (h,k,l)	−12 ≤ h < 12 −8 ≤ k < 8 −14 ≤ l < 14	−5 ≤ h < 7 −11 ≤ k < 11 −39 ≤ l < 39
Completet o2 (%)	2.575	2.868
Data/restraints/parameter	3396/1/235	3204/0/247
Goodness-of-fit (F^2^)	1.043	1.097
Absorption correction	None	None
Rindices [l > 2*σ*(1)]	0.1857	0.0939
Rindices (alldata)	0.0852	0.0829

S7:

The active component assigned as S7 was a colorless crystal. The deduced molecular formula was C_20_H_18_O_6_, mp.pt = 122 °C–125 °C. Mass: observed: 709.22 (dimer form); calculated: 354.34, m/z: 90.50, 115.05, 185.06, 319.09, 337.10, 387.19, 409.17, 486.29, 564.28, 709.22. ^13^C: ^δ^C: 54.23(C-1), 71.43(C-2), 85.34(C-3), 101.36(C-4), 107.01(C-5), 108.41 (C-6), 119.82 (C-7), 135.93(C-8), 146.97(C-9), 147.88. H-NMR, ^δ^H: 6.82–6.92 (m, 5H), 5.9 (s, 1H), 4.09–4.65 (m, 1H), 3.75 (s, 3H). Based on NMR and crystallography data, active component S7 was confirmed as sesamin. The ORTEP diagram is also drawn with 50% thermal ellipsoid in Figure 5E.

S8:

The active component assigned as S8 was also a colorless crystal. The deduced molecular formula was C_21_H_22_O_6_, mp.pt = 133 °C–136 °C. Mass: observed: 741.29 (dimer form); calculated: 370.38, m/z: 66.03, 135.04, 189.09, 225.56, 335.12, 353.13, 370.14, 390.12, 488.07, 575.19, 741.29. ^13^C: ^δ^C: 49.71(C-1), 54.43(C-2), 55.88(C-3), 55.97(C-4), 69.28(C-5), 70.71(C-6), 81.69(C-7), 87.17(C-8), 101.31(C-9), 106.65, 108.43, 110.14, 111.98, 118.68, 119.05, 133.09, 134.29, 146.44, 147.55,148.66, 149.18. H-NMR, ^δ^H: 6.82–6.92 (m, 5H), 6.0 (s, 2H), 4.7 (d, 1H), 4.3 (d, 1H), 4.0 (d, 1H), 3.7 (s, 6H), 3.3 (d, 1H), 3.0 (d, 1H) 2.8 (d, 1H). Based on NMR and crystallography data, active component S8 was confirmed as fargesin. The ORTEP diagram is also drawn with 50% thermal ellipsoid in Figure 5J.

### 4.6 Sesamin and fargesin inhibited inflammation on CpG-stimulated conventional type 1 dendritic cells

**FIGURE 6:**
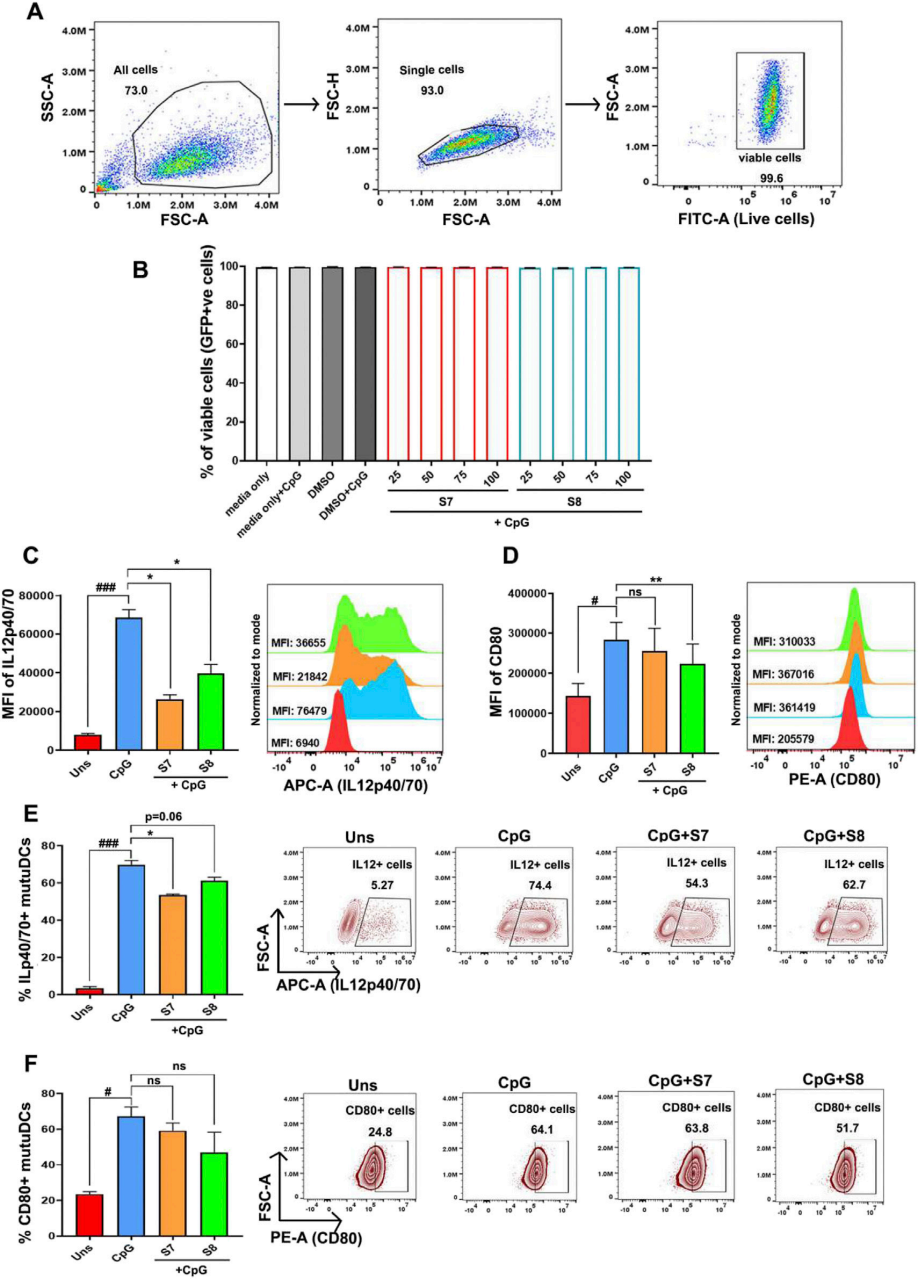
Anti-inflammatory activity of the bioactive compounds sesamin and fargesin on the protein expression levels of IL-12 and CD80 in CpG-stimulated cDC1 cells. The dendritic cells were pre-treated with 25 μg/mL concentrations of compounds for 1 h, and then, the cells were stimulated with 1 μg/mL of CpG for 6 h. **(A)** Flow cytometry gating strategy to measure GFP + cells (proxy to check viability). **(B)** The cell viability of normal and CpG-stimulated mutuDCs intervened with S7/S8 (n = 3) **(C)** Bar graph and histogram plot showing MFI (mean fluorescence intensity) levels and changes in intracellular IL12p40/70 secretion levels after 6 h in CpG-activated mutuDCs, before and after treatment with sesamin (S7) and fargesin (S8). Changes were measured for GFP^+^-gated mutuDCs by flow cytometry (n = 3). **(D)** Bar graph and histogram plot showing MFI levels and changes in CD80 surface expression levels after 6 h in CpG-activated mutuDCs, before and after treatment with sesamin (S7) and fargesin (S8). Changes were measured for GFP^+^-gated mutuDCs by flow cytometry (n = 3). **(E)** Bar graphs and scatterplots showing changes in percent positive cells, representative of intracellular IL12p40/70 secretion, after 6 h in CpG-activated mutuDCs, before and after treatment with sesamin (S7) and fargesin (S8). Changes were measured for GFP^+^-gated mutuDCs by flow cytometry (n = 3). **(F)** Bar graphs and zebra plots showing changes in percent positive cells, representative of surface CD80 expression, after 6 h in CpG-activated mutuDCs, before and after treatment with sesamin (S7) and fargesin (S8). Changes were measured for GFP^+^-gated mutuDCs by flow cytometry (n = 3). Each value expresses the mean ± SEM of triplicates, and # p-value <0.05 and ### p-value <0.001 of the CpG group were compared with those of the unstimulated group. *p-value <0.05 and **p-value <0.01 of the S7/S8-treated groups were compared to those of the only CpG-treated group. ns, not significant.

To investigate whether sesamin and fargesin directly act on dendritic cells, we conducted an *in vitro* study using MuTuDCs. We then established an inflammation model in mutuDCs using CpG, a ligand for Toll-like receptor 9 (TLR9). The cell viability was found to be intact upon treatment with sesamin and fargesin. cDC1 cells are generally known to secrete pro-inflammatory cytokines when exposed to CpG (Figures 6A,B). In this study, to confirm the anti-inflammatory effects of the compounds on the intracellular secretion of pro-inflammatory cytokines secreted from cDC1 cells, we investigated the inhibitory effect of sesamin and fargesin on the CpG-induced production of IL12, which plays a significant role in inflammation. MuTuDCs were pretreated with the bioactive compounds for 1 h and then exposed to CpG for 6 h. Flow cytometry analysis showed that compared with those in the unstimulated group, the levels of intracellular secretion of IL-12 were significantly elevated in the CpG group; moreover, application of the compounds significantly reduced the levels of IL-12 in the CpG group (Figure 6C). Furthermore, exposure of MuTuDCs to CpG led to an increase in the expression of CD80—a key cell surface marker to mark the activation level of inflammatory dendritic cells. It is noteworthy that the level of CD80 decreased significantly when the inflammatory cells were administered with fargesin, indicating that fargesin could inhibit the activation of dendritic cells. However, sesamin did not possess similar potential (Figure 6D).

We found a similar trend of reduction in intracellular IL12 in terms of the percent positive cells, upon S7/S8 treatment in inflammatory MutuDCs (Figure 6E). However, percent positive cells for CD80 were comparable between the CpG-treated group and the S7/S8-treated CpG-activated group (Figure 6F). Overall, sesamin and fargesin exert anti-inflammatory potentials through direct activity on pro-inflammatory dendritic cells.

### 4.7 ADMET analysis

*In silico* ADMET analysis and prediction of drug-likeness can reveal the safety requirements and bioavailability, which otherwise could have affected the tested animals. Moreover, the high cost of clinical trials could be avoided. The molecular properties, pharmacokinetics, toxicities, and drug-likeness profile of the isolated compounds (S7 = sesamin and S8 = fargesin) were predicted using the *in silico* ADMET analysis and the prediction tool ADMETlab 2.0, which is available online, as presented in Table 5.

**TABLE 5:** Molecular properties, predicted pharmacokinetic, toxicity, and drug-likeness profile of the isolated compounds (S7 = sesamin and S8 = fargesin).

Parameter	Calculated value (sesamin)	Calculated value (fargesin)	Optimal value
Physiochemical characteristic			
Molecular weight	354.35	370.14	100–600
logP	3.326	2.883	1–3
H-bond acceptor	6	6	0–12
H-bond donor	0	0	0–7
Rotatable bonds	2	4	0–11
Polar surface area	55.38	55.38	0–140
Absorption characteristic			
Caco-2	−4.904	−4.859	≥-5.15
MDCK	6.2e-05	7.5e-05	Medium: 2-20 × 10^-6^ cm/s High: >20 × 10^-6^ cm/s
Pgp-inhibitor	0.813	0.969	Inhibitor:1 Non-inhibitor:0
Pgp-substrate	0.0	0.001	Substrate:1 Non-substrate:0
HIA	0.001	0.001	HIA+: 1 HIA-: 0
F20%	0.004	0.005	F20%+: 1 F20%-: 0
F30%	0.042	0.06	F30%+: 1 F30%-:0
Distribution characteristic			
BBB	0.177	0.197	BBB+: 1 BBB−: 0
PPB	96.76	91.91	<90
VD	0.888	1.048	0.04–20L/kg
Fu	2.613	3.108	Middle: 5–20 High: >20
Metabolism characteristic			
CYP1A2 inhibitor	0.617	0.101	Inhibitor: 1 Non-inhibitor: 0
CYP1A2 substrate	0.314	0.942	Substrate: 1 Non-substrate: 0
CYP2C19 inhibitor	0.949	0.854	Inhibitor: 1 Non-inhibitor: 0
CYP2C19 substrate	0.211	0.812	Substrate: 1 Non-substrate: 0
CYP2C9 inhibitor	0.779	0.641	Inhibitor: 1 Non-inhibitor: 0
CYP2C9 substrate	0.778	0.794	Substrate: 1 Non-substrate: 0
CYP2D6 inhibitor	0.959	0.778	Inhibitor: 1 Non-inhibitor: 0
CYP2D6 substrate	0.917	0.925	Substrate: 1 Non-substrate: 0
CYP3A4 inhibitor	0.964	0.953	Inhibitor: 1 Non-inhibitor: 0
CYP3A4 substrate	0.511	0.78	Substrate: 1 Non-substrate: 0
Excretion characteristic			
CL	18.247	14.941	High: >15 mL/min/kg Medium: 5–15 mL/min/kg
T_1/2_	0.062	0.095	Long: 1 Short: 0
Toxicology characteristic			
Hepatotoxicity	0.24	0.185	HHT+: 1 HHT−: 0
DILI	0.889	0.903	DILI+: 1 DILI−: 0
Ames toxicity	0.755	0.449	Ames+: 1 Ames−: 0
ROA	0.153	0.056	ROA+: 1 ROA−: 0
Skin sensitization	0.728	0.78	Sensitizer+: 1 Sensitizer−: 0
Carcinogenicity	0.866	0.771	Carcinogen+: 1 Carcinogen−: 0
Eye corrosion	0.003	0.003	Corrosive: 1 Non-corrosive: 0
Respiratory toxicity	0.436	0.479	Respiratory toxicants: 1 Respiratory non-toxicants: 0
Drug-likeness characteristic			
QED	0.825	0.821	>0.67
SA	3.774	3.629	Easy to synthesize: <6 Difficult to synthesize: ≥6
Fsp3	0.4	0.429	≥0.42
PAINS	0	0	0 alerts
Lipinski	Accepted	Accepted	All in range
Pfizer	Rejected	Accepted	1 accepted, 1 rejected

HBAm, hydrogen bond acceptor; HBD, hydrogen bond donner; Pgp, phospho-glycoprotein; logp, logarithm of the partition coefficient; VD, volume distribution; BBB, blood–brain barrier; HIA, human intestinal absorption; F20%, oral bioavailability of 20%; F30%, oral bioavailability of 30%; PPB, plasma protein bound; Fu, fraction bound to plasma; CYP, human cytochrome P450 family; CL, clearance; T1/2, half-life; DILI, drug-induced liver injury; ROA, rat oral toxicity; TPSA, total polar surface area; QED, quantitative estimate of drug-likeness; SA, synthetic accessibility; Fsp3, fraction of sp3 carbon atoms; PAINS, potential pharmacokinetic and interaction scenarios.

The molecular weights of both the compounds fall within the preferable range for oral absorption; their hydrogen bond acceptors and donors (HBAs and HBDs), rotatable bonds, and polar surface areas are also in agreement. Both the compounds showed Caco-2 permeability and MDCK permeability; both of them showed excellent Pgp inhibitor scores and better oral bioavailability.

Both compounds show agreement in volume distribution (VD) and exhibit moderate blood–brain barrier (BBB) permeability but display high plasma protein binding and a lower fraction bound to plasma (Fu).

For the five isozymes of the human cytochrome P450 family, all values fall between 0 and 1. Regarding excretion, sesamin scores high and fargesin scores medium in clearance (CL), with both showing excellent half-lives (T_1/2_). Toxicity values are higher for drug-induced liver damage, skin sensitization, and carcinogenicity, whereas other toxicity scores are lower for both the compounds.

**FIGURE 7:**
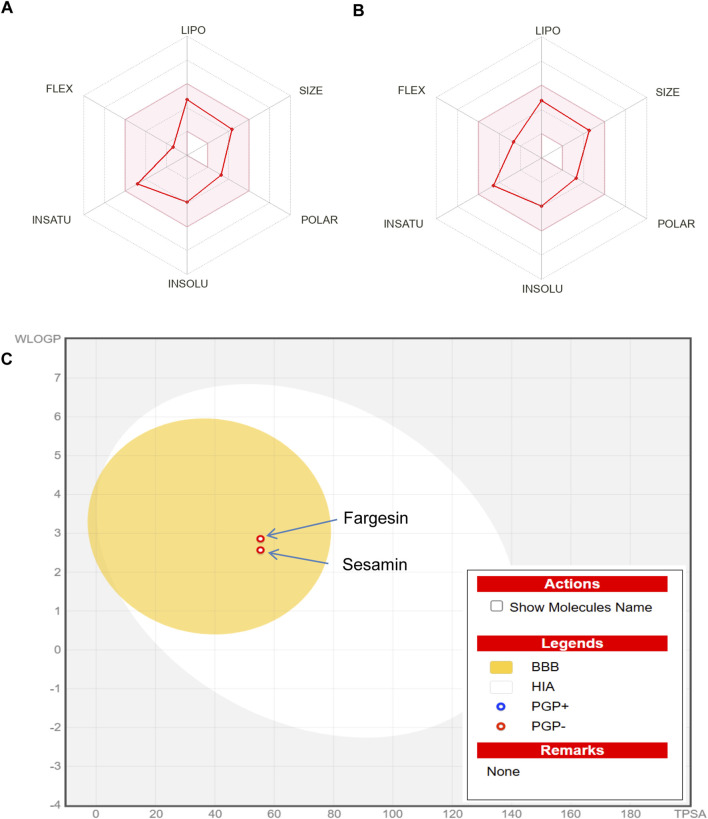
**(A)** Bioavailability radar chart for sesamin. **(B)** Bioavailability radar chart for fargesin. **(C)** swissADME prediction of boiled-egg plot for the isolated compounds sesamin and fargesin. The yellow area represents the blood–brain barrier penetration, and the white area represents the gastro-intestinal absorption. BBB, blood–brain barrier penetration; HIA, human intestinal absorption.

Both the compounds complied with the Lipinski drug-likeness test as molecular weight ≤500; logP ≤5; HBA ≤10; HBD ≤5 but rejected the compound sesamin by Pfizer as logP> 3; TPSA <75. Compounds with a high log P (>3) and low TPSA (<75) are likely to be toxic (Table 5). Boiled-egg plot using the SwissADME server showed favorable blood–brain barrier penetration and gastro-intestinal absorption for both the compounds (Figure 7).

## 5 Discussion

Evidence-based validation of folk medicine claims through modern scientific techniques, along with the identification of their phytochemical and toxicity profiles, is essential to fully explore the therapeutic importance of medicinal plants. In the current study, *Z. armatum* DC. was selected based on previous studies documenting the crude extract’s biological activities and therapeutic potential (Singh et al., 2022; Sati et al., 2011). In this study, the crude hydromethanolic extract (ZS) of the stem of *Z. armatum* DC. was taken for bioassay-guided fractionation into four different fractions in hexane (ZSH), ethyl acetate (ZSE), butanol (ZSB), and methanol (ZSM), enabling the separation of potential bioactive compounds. In preliminary phytochemical analysis, the plant extract ZS was detected to be rich in phenolics and flavonoids, which are key secondary metabolites of plants and considered natural anti-inflammatory agents. Quantitative evaluation of the different fractions showed the highest amount of phenolics (100.00 µg GAE/mg dried extract) and flavonoids (101.33 µg QE/mg dried extract) in the ZSE, followed by ZSH, ZSB, and ZSM (Supplementary Table S5). The amounts of both phenolic and flavonoid were found remarkably higher in *Z. armatum* DC., indicating this plant to be a potent source of polyphenols.

We focused on the anti-inflammatory activity of different bioactive fractions of hydromethanolic extracts of the *Z. armatum* DC. stem using protein-based or cell-based assays. Such assays help minimize the ethical issues related with animal usage during the initial stages of drug discovery and get an idea about possible mechanism of action(s) of phytoconstituents. In this study, human RBC membrane stabilization assay and protein anti-denaturation assay were conducted, which are considered to be useful screening platforms (Akhtar, 2022) as these methods are simple, rapid, and reproducible. The RBC membrane is structurally analogous to the lysosome membrane. During inflammation, the lysosome membranes are ruptured, releasing bactericidal enzymes and proteases from the lysosomes of those damaged tissues, which can further cause healthy tissue damage (Sonter et al., 2021). Bioactive compounds/plant extracts with the potential to safeguard RBC from membrane rupture are, therefore, expected to inhibit the leakage of inflammatory substances from lysosomes through the stabilization of the lysosome membrane and further be correlated with their anti-inflammatory potential (Ezzat et al., 2018). In the hemolysis assay, the results revealed that only ZSE among four of the sequential extracts of ZS was capable to inhibit RBC lysis, with IC_50_ of 56.00 ± 0.00 μg/mL compared with IC_50_ of 58.86 ± 0.11 μg/mL of diclofenac sodium (Figure 4A). Tissue protein denaturation is also considered one of the well-documented markers of inflammation as those damaged tissues generate autoantigens in certain inflammatory diseases, for example, arthritic diseases (Ezzat et al., 2018). Several physical and chemical factors, including heat, stress, and chemical exposure, lead to denaturation of native structure of proteins, a hallmark of autoantigen production (Saleem et al., 2020). Hence, any extract/bioactive compound possessing the protein anti-denaturation property can be considered to have anti-inflammatory potential. In the current study, the results revealed that ZSE exhibited remarkable anti-inflammatory activity by significantly inhibiting protein denaturation compared to diclofenac. ZSE showed IC_50_ of 45.00 ± 0.00 μg/mL compared with IC_50_ of 47.90 ± 0.15 μg/mL of diclofenac sodium (Figure 4C).

The GC–MS analysis of the hydromethanolic stem extract and its ethyl acetate fraction detected 71 and 78 compounds, respectively. Among them, 19 compounds from the hydromethanolic stem extract and 17 compounds from the ethyl acetate fraction were considered the major compounds (>0.5% relative area percentage). Six major compounds with >5% are present in both the hydromethanolic stem extract and its ethyl acetate fraction. A furo-furan lignan, fargesin, has the highest concentration in both the hydromethanolic stem extract and its ethyl acetate fraction, with percentage areas of 26.49% in the hydromethanolic extract and 27.88% in the ethyl acetate fraction. Lignans constituted 55.79% and 58.22% of the total percent area concentration in the extract and ethyl acetate fraction, respectively. Singh et al. (2020), for the first time, reported GC–MS analysis of chloroform and methanol extracts of *Z. armatum* DC. leaf and bark. In that study, 56 compounds in the methanolic extract and 82 compounds in the chloroform extract were reported from the bark. Butylamine (23.1%) has the highest concentration in the methanolic extract, and yangambin (2.4%) was one of the major compounds detected in the chloroform extract. However, 17 major compounds in the methanolic extract and 15 major compounds in its ethyl acetate fraction detected in our GC–MS analysis are different from those identified by Singh et al., 2020. Seasonal variations in the volatile phytochemical composition of the stem of *Z. armatum* DC. were analyzed by Devi et al., 2023. In that study, 25 compounds were identified in the winter sample, 22 in the spring sample, 22 in the summer sample, and 16 in the monsoon sample. However, all 19 major compounds in the methanolic extract and all 17 major compounds in its ethyl acetate fraction detected in our study are different from those identified by Devi et al. (2023). Two compounds detected in that study, 2-undecanone and caryophyllene, were found in trace amounts in our study.

In this study, we reported to isolate two known compounds, sesamin and fargesin, from the ethyl acetate fraction as it exhibited the highest potency against protein denaturation and hemolysis. The structures of both compounds were determined using single crystal X-ray diffractometry and spectroscopic data (Figure 5) and compared with those in the previously reported literature. Both the compounds belong to the furo-furan lignan type, with a bicyclic oxygen structure, and have a multitude of bioactive potential including antioxidant, anticancer, anti-inflammatory, and neuroprotective effects (Osmakov et al., 2022). Both sesamin and fargesin showed remarkable inhibition potential during *in vitro* anti-inflammatory assays. In the heat-induced hemolysis inhibition assay, sesamin showed IC50 of 45.09 ± 0.17 SD ug/mL and fargesin showed 40.24 ± 0.01 SD ug/mL (Figure 4A), which are lower than that of the standard anti-inflammatory drug, diclofenac (58.86 ± 0.11 SD ug/mL). In the heat-induced albumin denaturation inhibition assay, sesamin showed IC50 of 31.38 ± 0.55 SD ug/mL and fargesin showed 42.60 ± 0.14 SD ug/mL (Figure 4C), which are also lower than that of diclofenac (47.90 ± 0.15 SD ug/mL).

In the existing literature, both sesamin and fargesin had been well documented for their anti-inflammatory bioactivities. Pham et al. (2017) reported that fargesin exhibited anti-inflammatory potential in human macrophages by suppressing the key signal transduction enzyme, the protein kinase C (PKC) pathway, including downstream c-Jun N-terminal kinase (JNK), nuclear factor activator protein-1 (AP-1), and nuclear factor-kappa B (NF-ĸB). In 2018, for the first time, Yue et al. (2018) demonstrated the *in vivo* anti-colitic properties of fargesin using a murine colitis model, which was associated with NF-κB pathway downregulation. In another report, it was demonstrated that fargesin could mitigate atherosclerosis through reduction in vascular inflammation (Wang et al., 2020). In the setting of osteoarthritis, fargesin attenuated disease progression by influencing the switching of macrophages from pro-inflammatory to anti-inflammatory phenotype and by suppressing mitogen-activated protein kinase (MAPK) and NF-κB signaling. Zhang et al. (2022) showed that fargesin has potential to suppress the inflammatory response, which further contributes to the attenuation of lung injury. In addition, sesamin was reported to inhibit lipopolysaccharide-induced cytokine production by suppressing NF-κB activation and the MAPK signal pathway (Jeng et al., 2005). In a recent study, Abe-Kanoh et al. (2019) reported that sesamin exerted anti-inflammatory effects in inflammatory mouse macrophages via suppression of interferon-β and inducible nitric oxide synthase expression. In a murine model of chemically induced colitis, sesamin was shown to mitigate damage of colon by suppressing the NF-κB and MAPK signaling pathways, exerting anti-inflammatory effects *in vivo* (Chen et al., 2021).

Dendritic cells are considered “professional” antigen-presenting cells characterized by their high expression of co-stimulatory molecules (i.e., CD80, CD86, and MHC II) on their surface. These co-stimulatory molecules engage with naive T cells and initiate their activation and differentiation (Steinman, 1991; Young et al., 1992). Notably, fargesin significantly inhibited the expression of one of the key co-stimulatory molecules on cDC1s (Figure 4D), indicating that fargesin can repress the function of these immune cells in the differentiation of naive T cells. In addition, cytokines also play crucial roles in maintaining the immune system. In this context, IL-12, a key pro-inflammatory cytokine produced by cDC1 cells, is important in inducing the development and growth of Th1 cells, a subset of CD4^+^ T helper cells (Blanco et al., 2008). Our results further support the previous findings of anti-inflammatory potential of sesamin and fargesin by demonstrating that both of these compounds significantly reduce the level of pro-inflammatory cytokine (IL-12) in CpG-stimulated cDC1s (Figure 4C). This suggests that *Z. armatum* DC. may exert anti-inflammatory effects by direct modulation of cDC1 activation and pro-inflammatory cytokine production through the activity of sesamin and fargesin, which offers a novel lead into the mechanism of action of *Z. armatum* DC.

Predictions on accurate ADMET properties are very crucial for determining the potential of a bioactive molecule for paving its way toward drug development. It is estimated that approximately 40% of drugs fail during the course of drug discovery due to a lack of the pharmacokinetic profile. The predicted data for sesamin and fargesin on pharmacokinetic analysis, bioavailability radar, and drug-likeness supported the suitability of both the bioactive compounds for oral administration (Figure 7) and acknowledged their feasibility to be safer and promising agents for future therapeutic drug development.

Sesamin and fargesin are promising candidates, particularly given their ability to inhibit the co-stimulatory molecules and pro-inflammatory cytokine production in cDC1 cells, which strongly supports their potential for therapeutic development. Their ability to suppress IL-12 production by cDC1 cells further positions them as valuable leads for modulating adaptive immunity, potentially attenuating Th1/Th17-mediated inflammatory conditions. From a translational perspective, both sesamin and fargesin meet several criteria that would be preferred in drug development. Predictions of *in silico* ADMET characteristics indicated a favorable oral bioavailability profile, drug-likeness properties, and safety profile, which are typically key bottlenecks in early drug development. Scalability and cost-effective sourcing are also facilitated by their natural presence in *Zanthoxylum armatum* DC. In addition, these lignans, compared to the traditional NSAIDs and SAIDs, seem to be multi-targeted with potentially reduced side effects because they do not promote cytotoxicity in cDC1 cells but suppress immune activation. This highlights their potential as next-generation, plant-based immunomodulators or adjunct therapies.

Formulations such as nano-encapsulation or liposomal delivery may enhance their stability, bioavailability, and targeted tissue delivery. In combination therapy, particularly as adjuvants, they may reduce SAID and NSAID dosages and should also be explored. The anti-inflammatory and antioxidant properties of both compounds make them promising candidates not only for inflammatory diseases but also for metabolic syndromes, neurodegeneration, and even cancer, where inflammation is a key pathological component. Thus, sesamin and fargesin hold substantial potential for translation into safe, plant-based therapeutic agents for a broad spectrum of inflammatory and immune-related disorders.

## 6 Limitations and future directions

The study has several limitations. First, this study was designed to identify the anti-inflammatory bioactive compound(s) and provide scientific evidence of the folklore uses of *Z. armatum* DC. against inflammation. However, *in vivo* studies were not included due to the lack of proper infrastructure to substantiate the anti-inflammatory potential and safety of the compounds in a suitable animal model. Second, the results identified two bioactive compounds with promising pharmacokinetic analysis, bioavailability radar, and drug-likeness suitability. However, the pharmacokinetic and toxicity profiles were based on computational predictions, which are predictive in nature and not experimentally validated due to infrastructural limitations.

Future directions of this study include the following: first, to explore detailed molecular mechanisms by which sesamin and fargesin modulate inflammatory pathways in dendritic cell signaling and second, to conduct *in vivo* experiments to validate the anti-inflammatory efficacy and safety of sesamin and fargesin in a suitable animal model.

## 7 Conclusion

Taken together, in this study, we support the ethnopharmacological relevance of *Z. armatum* DC. by validating its traditional therapeutic use and contributing to preclinical stages of the drug discovery process through anti-inflammatory evaluations of different sequential fractions, isolation of bioactive compounds, and evaluation of their effects on cDC1 cells. The bioassay-guided examination on the hydromethanolic stem extract of *Z. armatum* DC. resulted in the isolation and identification of sesamin and fargesin, two known lignans. The results revealed that both the compounds were mainly responsible for the anti-inflammatory effect of the ethyl acetate fraction hydromethanolic stem extract of *Z. armatum* DC. determined through the albumin denaturation inhibition assay and heat-induced hemolysis inhibition assay. To the best of our knowledge, this is the first study reporting an anti-inflammatory activity of sesamin and fargesin against pro-inflammatory conventional type 1 dendritic cells as it was able to inhibit the production of a key pro-inflammatory cytokine analyzed herein. The findings justified the conventional utilization of *Z. armatum* DC. and suggested that its stem extract could be developed to be a promising therapeutic agent to alleviate inflammatory diseases. From a future perspective, well-designed *in silico* studies, supported by different *in vivo* models of inflammatory diseases, are recommended to understand the benefits of *Z. armatum* DC.

## Data Availability

The original contributions presented in the study are included in the article/Supplementary Material; further inquiries can be directed to the corresponding author.

## Glossary

ADMET: Absorption, distribution, metabolism, excretion, and toxicity
AIRF: Advanced Instrumentation Research Facility
AP-1: Activator protein-1
BBB: Blood–brain barrier
Caco-2: Cancer coli-2 cell line
**CD4** ^**−**^: Cluster of differentiation 4
**CD80** ^**−**^: Cluster of differentiation 80
**CD86** ^**−**^: Cluster of differentiation 86
cDC1: Conventional type 1 dendritic cells
CIF: Central Instrumentation Facility
CL: Clearance
CO2: Carbon dioxide
CpG: Cytosine phosphorothioate guanine oligodeoxynucleotides
CYP: Human cytochrome P450 family
DILI: Drug-induced liver injury
DMSO: Dimethyl sulfoxide
Dual AJS ESI: Dual Agilent Jet Stream Electrospray Ionization
F20%: Oral bioavailability of 20%
F30%: Oral bioavailability of 30%
FACS: Fluorescence-activated cell sorting buffer
FBS: Fetal bovine serum
FFNSC: Mass Spectra of Flavors and Fragrances of Natural and Synthetic Compounds
Fsp3: Fraction of sp3 carbon atoms
Fu: Fraction bound to plasma
GAE: Gallic acid equivalent
GC–MS: Gas chromatography–mass spectrometry
GFP: Green fluorescent protein
HBA: Hydrogen bond acceptor
HBD: Hydrogen bond doner
HCl: Hydrochloric acid
HEPES: N-(2-Hydroxyethyl)piperazine-N'-(2-ethanesulfonic acid)
HIA: Human intestinal absorption
HPLC: High-performance liquid chromatography
HRMS: High-resolution mass spectrometry
IBSD: Institute of Bioresources and Sustainable Development
IC50: Half-maximal inhibitory concentration
IIT: Indian Institute of Technology
IL12: Interleukin 12
IMDM: Incomplete Iscove’s modified Dulbecco’s medium
JNK: c-Jun N-terminal kinase
LC–MS: Liquid chromatography–mass spectrometry
logp: Logarithm of the partition coefficient
MAPK: Mitogen-activated protein kinase
MDCK: Madin–Darby canine kidney cell line
MHC II: Major histocompatibility complex class II
NF-kB: Nuclear factor-kappa B
NIST: National Institute of Standard and Technology
NMR: Nuclear magnetic resonance
NSAIDs: Nonsteroidal anti-inflammatory drugs
ORTEP: Oak Ridge Thermal Ellipsoid Plot Program
PAINS: Potential pharmacokinetic and interaction scenario
PBS: Phosphate buffer saline
PDA: Photodiode array
Pgp: Phospho-glycoprotein
PKC: Protein kinase C
PPB: Plasma protein bound
QE: Quercetin equivalent
QED: Quantitative estimate of drug-likeness
Q-TOF: Quadrupole time-of-flight
RBC: Red blood corpuscle
ROA: Rat oral toxicity
SA: Synthetic accessibility
SAIDs: Steroidal anti-inflammatory drugs
SAIF: Sophisticated Analytical Instrument Facility
SARS COVID-19: Severe acute respiratory syndrome corona virus disease 2019
T1/2: Half-life
TFC: Total flavonoid content
Th1: T-helper 1 cells
TLR9: Toll-like receptor 9
TPC: Total phenolic content
TPSA: Total polar surface area
UPLC: Ultra-performance liquid chromatography
VD: Volume distribution
WHO: World Health Organization
XRD: X-ray diffractometry
ZS: *Zanthoxylum armatum* DC. hydromethanolic stem extract
ZSB: Butanol fraction of the *Zanthoxylum armatum* DC. hydromethanolic stem extract
ZSE: Ethyl acetate fraction of the *Zanthoxylum armatum* DC. hydromethanolic stem extract
ZSH: Hexane fraction of the *Zanthoxylum armatum* DC. hydromethanolic stem extract
ZSM: Methanol fraction of the *Zanthoxylum armatum* DC. hydromethanolic stem extract
