# Supplementary material for: Bioassay-guided isolation of sesamin and fargesin from the hydroalcoholic stem extract of Zanthoxylum armatum DC. inhibited inflammation in CpG-stimulated conventional type 1 dendritic cells
Source: Front Pharmacol. 2025 Nov 7;16:1687789. doi: 10.3389/fphar.2025.1687789 (PMC12634626; doi:10.3389/fphar.2025.1687789)

**Supplementary Information**

**Taxonomic Classification of *Zanthoxylum armatum* DC.:**

Kingdom: Plantae

Class: Magnoliopsida

Order: Rutales

Family: Rutaceae

Genus: *Zanthoxylum*

Binomial Name: *Zanthoxylum armatum*

**Common Names:**

Manipuri: Mukthrubi

Sanskrit: Tumbru, dhiva, ghndhalu, Tejovha, Saurabha, Tejovati, Tumbaru, Vanaja

Hindi: Dharmar, tejphal, Trimal, Nepali dhaniya

Mizoram: Arhrikreh

Assamese: Kulekhara

Bengali: Gaira, tambal, Tejovati

Oriya: Tundopoda, Arhrikreh, Ranabelli

Gujrati: Tejabala,Tejbal

Kannada: Tejapatri, Jimmi, Tumbura,Tumburudra,Tejovanti, Dhiva

Malayalam: Thumboonal, Thumbooni,Valiyavaluzhavam

Marathi: Tejabal, Chirphal, Naepaalidhane

Tamil: Thejyovathi, Tumpunalu

Telugu: Gandhalu, Konda-kasimi, Konda, Kaasimanda

Urdu: Kabab-e-Khandan

Nepali: Timur

Tibetan: Gyer-ma

Burmese: Gawra Kha Nan Nan, Teza Bo

Chinese: Ci Zhu Ye Hua Jiao, Qin Jiao (Taiwan), Zhu Ye Jiao

Japanese: Fuyu Zanshou, Fuyu-Sansh

Korean: Gae San Cho

Thai: Mak Kak

German: Nepal pfeffer

English: Bamboo-Leaved Prickly Ash, Nepal Pepper, Prickly Ash, Prickly Ash bark, Toothache Tree, Winged Prickly Ash, Winged Prickly-Ash, Prickly Ash

**Preliminary Phytochemical Screening**

Standard qualitative and quantitative phytochemical analysis of the extracts were carried out for the various plant constituents and screened for their presence or absence by using standard procedures.

1. **Test for alkaloids:**
   1. **Dragendorff’s test**

To 3ml of extracts 4 drops of Dragendorff’s reagent were added. Formation of brownish red precipitates were observed indicating the presence of alkaloids.

- 1. **Hager’s Test**

To 3ml of extracts 4 drops of hager’s reagent were added. Bright yellow precipitates were formed confirming the presence of alkaloids.

1. **Test for flavonoids:**
   1. **Shinoda test**

To 3mL of extracts, 4 drops of concentrated hydrochloric acid and a small piece of magnesium turnings were added. Formation of pink color indicated the presence of flavonoids.

- 1. **Alkaline test**

To 1mL extracts, 2mL of 2% NaOH solution was added. Formation of yellow color which becomes colorless on addition of a dilute acid indicated the presence of flavonoids.

1. **Test for phenolic compounds:**
   1. **Lead acetate test**

To 1mL of extracts, 4 drops of 10% lead acetate solution were added. Formation of white precipitate indicated the presence of phenolic compounds.

- 1. **Ferric chloride test**

To 1ml of extracts, 4 drops 10% ferric chloride solution were added. Formation of dark green coloration indicated the presence of phenolic compounds.

1. **Test for carbohydrates:**
   1. **Molisch’s test**

To 3ml of extracts 4 drops of Molisch’s reagent were added and 1ml of conc. sulphuric acid was added along the side of the test tubes. A violet rings formed at the junction of the two liquid layers indicated the presence of carbohydrates.

- 1. **Test for reducing sugars:**

**2.2.a. Fehling’s test**

To 1ml of extracts, 1mL of Fehling’s solution A and 1mL of Fehling’s solution B were added and boiled in a water bath for 5 minutes. Formation of red precipitate indicated the presence of reducing sugars.

- 1. **Test for non-reducing sugars:**

To 3mL of extracts, 4 drops of dilute iodine solution were added. Formation of blue color but disappeared on boiling and reappeared on cooling indicated the presence of non-reducing polysaccharides.

1. **Tests for proteins and amino acids:**
   1. **Biuret test**

To 3mL of extracts, 4 drops of 1% copper sulphate solution and 1mL of 4% sodium hydroxide solution were added. Formation of pink coloration indicated the presence of proteins.

- 1. **Ninhydrin test**

To 3ml of extracts, 4 drops of 2% ninhydrin solution was added and kept at water bath for 5 minutes. Formation of deep blue coloration indicated the presence of amino acids.

1. **Test for tannins:**
   1. **Ferric chloride test**

To 3ml of extracts, 4 drops 10% ferric chloride solution were added. Formation of deep blue coloration indicated the presence of tannins.

1. **Test for steroids:**
   1. **Salkowski’s Test**

To 2ml of extracts, 2mL of chloroform and 2mL of concentrated sulphuric acid were added and shaken well. Formation of red coloration in chloroform layer and greenish yellow fluorescence in acid layer indicated the presence of steroids.

1. **Test for terpenoids:**
   1. **Triterpenoids**

To 3mL of extracts, 1mL of concentrated sulphuric acid was added and shaken well. Formation of a golden yellow layer at the bottom indicated the presence of triterpenoids.

- 1. **Diterpenoids**

To 3ml of extracts, 4 drops of copper acetate solution were added. Formation of an dark green coloration indicated the presence of diterpenoids.

1. **Test for glycosides:**
   1. **Test for cardiac glycosides**

**9.1. Keller-Kelliani test**

To 3mL of extracts, 2mL of glacial acetic acid and 4 drops of 5% ferric chloride were added and 1mL of concentrated sulphuric acid was added slowly along the sides of the test tubes. Appearance of reddish-browncolor at the junction of the two liquid layers and bluish green coloration of the upper layer indicated the presence of cardiac glycosides.

- 1. **Test for anthraquinone glycosides:**

To 3mL of extracts, 1mL of 10% ammonia solution was added. Formation of pinkish ammonical layer indicated the presence of anthraquinone glycosides.

- 1. **Test for saponin glycosides:**

To 3mL of extracts, 1mL of water was added and shaken vigorously in a test tube. Formation of persistent foam indicated the presence of saponin glycosides.

Supplementary Table 1: Instrument conditions for Gas Chromatography-Mass Spectrometry

| Item | Conditions |
| --- | --- |
| Detector | Thermal Desorption System TD 20 |
| Injector | Auto-injector system AOC-2oi |
| Sampler | Auto-sampler unit AOC-2os |
| Column | Rtx-5 MS (30m x 250µm, 0.25µm film thickness) |
| Temperature | 50ºC (5min) - 280ºC, at 5ºC/min, 69KPa |
| Gas flow | 3mL/min |
| Injection volume | 1µL |
| Split ratio | 1:10 |
| Injection/Detection temperature | 260ºC/270ºC |
| Flow rate | 1.21mL/min |
| Mass temperature | 50ºC (2min) to 210ºC (8min), at 3ºC/min, and finally upto 280ºC, at 8ºC/min |
| Ion source temperature | 230ºC |
| Interface temperature | 270ºC |
| Cut time | 2.50min |
| Threshold | 1000 |
| Scan speed | 3333amu/sec |
| Scan range | 40-650m/z |
| Total run length | 74min |
|  |  |

**Supplementary Table 2. HPLC peak table of isolated phyto-components from ethyl acetate fraction of hydromethanolic stem extract of *Zanthoxylum armatum* DC.S7= phyto-component isolated from active sub-fraction ZSE7, S8= phyto-component isolated from active sub-fraction ZSE8.**

| Sample code | Retention time | UV range |
| --- | --- | --- |
| S7 | 13.271 | 359nm |
| S8 | 26.662 | 237nm |

**Supplementary Table 3. Preliminary phytochemical screening of hydromethanolic extract of *Zanthoxylum armatum* DC. stem.**

| Sl. No. | Tests conducted for phytochemical constituents | Hydromethanolic extract of *Z armatum* DC. stem |
| --- | --- | --- |
| 1. | Alkaloids | |
|  | Dragendorff’s test | + |
|  | Hager’s test | + |
| 2. | Flavonoids | |
|  | Shinoda test | + |
|  | Alkaline test | + |
| 3. | Phenolics | |
|  | Lead acetate test | + |
|  | Ferric chloride test | + |
| 4. | Carbohydrates | |
|  | Molisch’s test | - |
|  | Reducing sugars | - |
|  | Non-reducing sugars | - |
| 5. | Proteins and amino acids | |
|  | Biuret test | - |
|  | Ninhydrin test | - |
| 6. | Tannins | |
|  | Ferric chloride test | + |
| 7. | Steroids | |
|  | Salkowski’s Test | - |
| 8. | Terpenoids | |
|  | Triterpenoids | - |
|  | Diterpenoids | + |
| 9. | Glycosides | |
|  | Cardiac glycosides | - |
|  | Anthraquinone glycosides | - |
|  | Saponin glycosides | + |

‘+’- presence, ‘-’- absence of tested phytochemicals.

**Supplementary Table 4. GCMS based analysis of relative percentages of major chemical components found in hydromethanolic stem extract of *Zanthoxylum armatum* DC. and its ethyl acetate fraction.**

| Components | R.time in ZS | R.time in ZSE | Area% in ZS | Area% in ZSE | Mol. Formula | Mol. Weight |
| --- | --- | --- | --- | --- | --- | --- |
| Dihydromaltol(1) | 8.40 | - | 0.78 | - | C6H8O4 | 144 |
| 5-Hydroxymethylfurfural(2) | 10.13 | - | 1.59 | - | C6H6O3 | 126 |
| Monoacetin(3) | 10.52 | - | 0.51 | - | C5H10O4 | 134 |
| Quinic acid(4) | 18.25 | - | 1.29 | - | C7H12O6 | 192 |
| (E)-Conipheryl alcohol(5) | 19.25 | 19.18 | 1.27 | 0.71 | C10H12O3 | 180 |
| Hexadecanoic acid(6) | 21.99 | 21.99 | 6.20 | 7.68 | C16H32O2 | 256 |
| Octadecanoic acid(7) | 23.78 | 23.78 | 10.50 | 10.38 | C18H36O2 | 284 |
| Heptacosanol(8) | - | 29.38 | - | 1.95 | C27H56O | 396 |
| n-Acetylanonaine(9) | 35.98 | 35.95 | 0.96 | 1.03 | C19H17NO3 | 307 |
| Butylamine(10) | 36.28 | 36.84 | 3.13 | 0.7 | C4H11N | 73 |
| 2,6-bis(3,4-methylenedioxyphenyl)-3,7-dioxybicyclo(3.3.0)octane(11) | 38.39 | 38.38 | 11.28 | 11.38 | C20H18O6 | 354 |
| Fargsin(12) | 39.23 | 39.22 | 26.49 | 27.88 | C21H22O6 | 370 |
| 1H,3H-Furo[3,4-c]furan,1,4-bis(3,4-dimethoxyphenyl)tetrahedro-,[1R-(1.alpha.,3a.beta.,4.alpha.,6a.alpha.)](13) | 40.53 | 40.74 | 13.87 | 14.42 | C22H26O6 | 386 |
| gamma-Sitosterol(14) | 42.25 | 42.25 | 3.24 | 4.7 | C29H50O | 414 |
| beta-Amyrin(15) | 43.21 | 43.19 | 1.08 | 0.84 | C32H52O2 | 468 |
| (3R,4R)-4-(3,4-dimethoxybenzyl)-3-(3,4,5-trimethoxybenzyl)dihydrofuran-2(3H)-one(16) | 43.41 | 43.37 | 0.89 | 0.69 | C23H28O7 | 416 |
| gamma-Tocopherol(17) | 44.04 | 43.99 | 5.37 | 5.63 | C30H50O3 | 458 |
| alpha-Amyrin(18) | 44.55 | 44.54 | 1.52 | 2.04 | C30H50O | 426 |
| 4-((2R,3R,4R,5R(-3,4-Dimethyl-5-(3,4,5-trimethoxyphenyl)tetrahydrofuran-2-yl)-2-methoxyphenol(19) | 45.79 | 45.74 | 1.23 | 0.73 | C22H28O6 | 388 |
| Isogmelinol(20) | - | 46.29 | - | 1.08 | C22H26O7 | 402 |
| Yangambin(21) | 48.06 | 48.03 | 2.03 | 2.04 | C24H30O8 | 446 |

Minimum concentration of chemical components in Area% ≥ 0.5.

Total % of chemical components in hydromethanolic stem extract (ZS) =93.23.

Total % of chemical components in ethyl acetate fraction of stem extract (ZSE) =93.88.

% contribution of lignans in total % of chemical components in hydromethanolic stem extract (ZS) =55.79.

% contribution of lignans in total % of chemical components in ethyl acetate fraction (ZSE) =58.22.

**Supplementary Figure 1:** Total phenolic content (TPC) and total flavonoid content (TFC) estimation. (A) Standard calibration curve of gallic acid. (B) Standard calibration curve of quercetin. (C) Total phenolic content in µg GAE/mg of dry weight of hydromethanolic stem extract and its four fractions of *Zanthoxylum armatum* DC. (D) Total flavonoid content in µg QE/mg of dry weight of hydromethanolic stem extract and its four fractions of *Zanthoxylum armatum* DC. ZSH- hexane fraction of ZS, ZSE- ethyl acetate fraction of ZS, ZSB- butanol fraction of ZS and ZSM- methanol fraction of ZS.


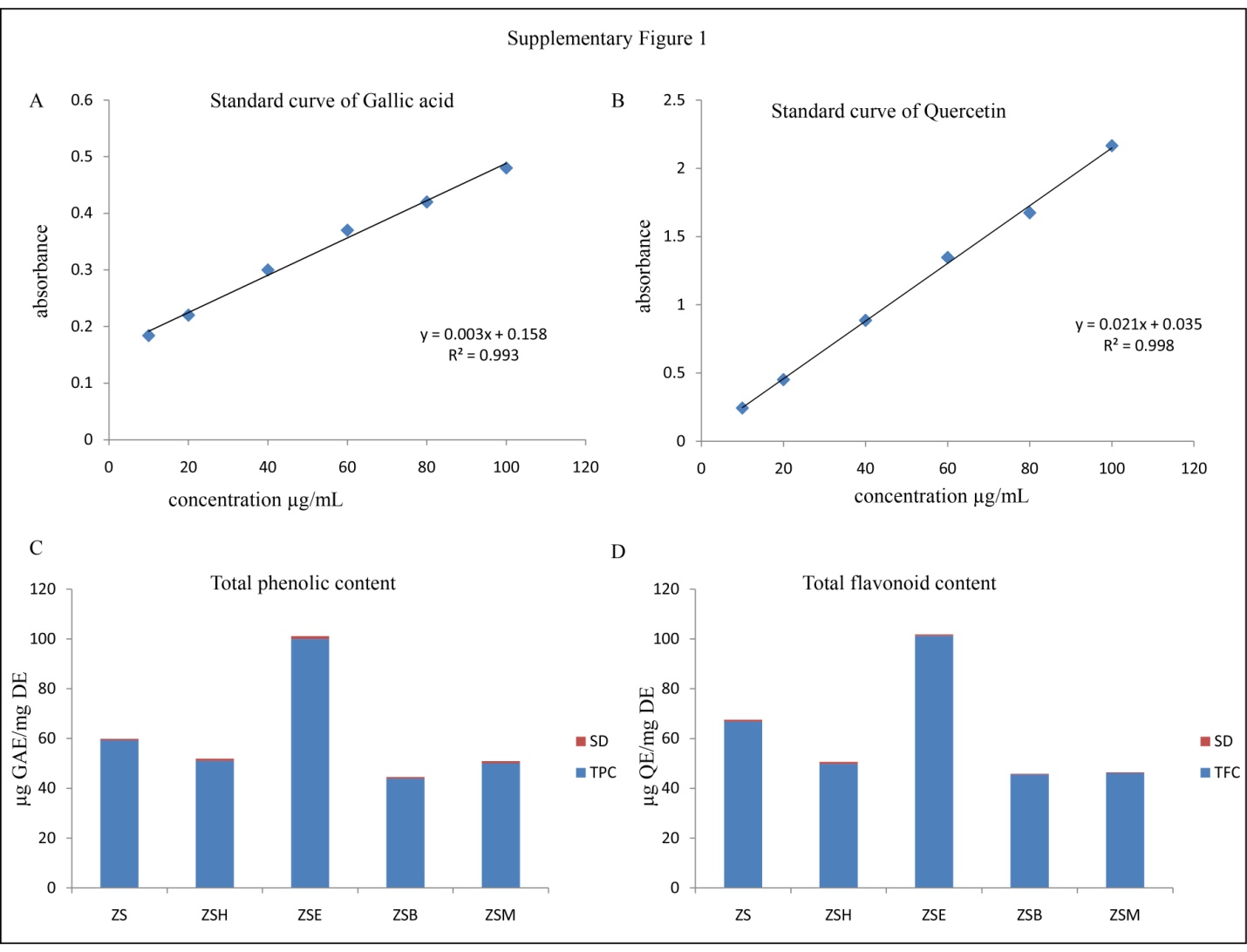


**Supplementary figure 2.** COSY spectra of isolated phyto-component S7 from ethyl acetate fraction of hydromethanolic stem extract of *Zanthoxylum armatum* DC.


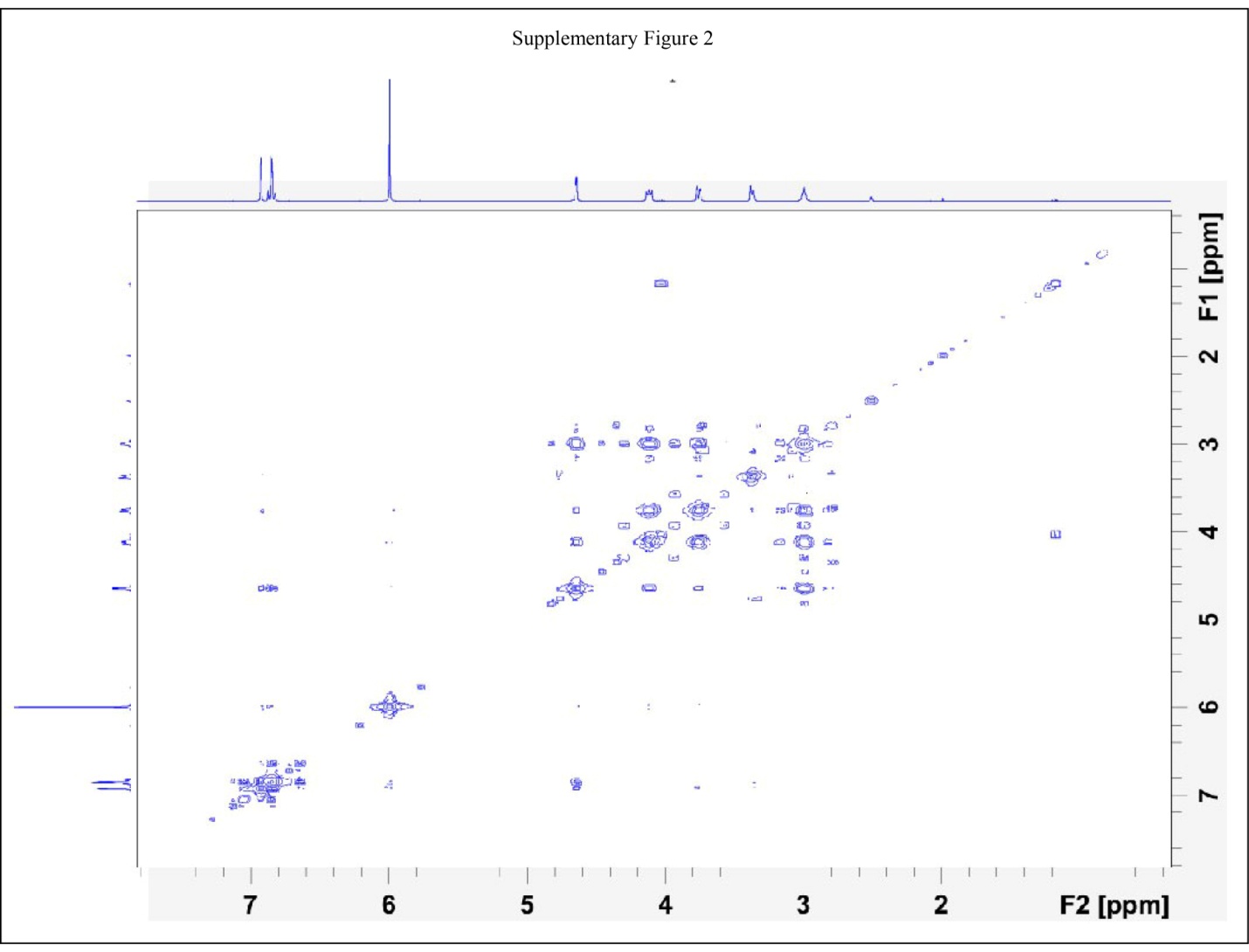


**Supplementary figure 3.** NOESY spectra of isolated phyto-component S7 from ethyl acetate fraction of hydromethanolic stem extract of *Zanthoxylum armatum* DC.


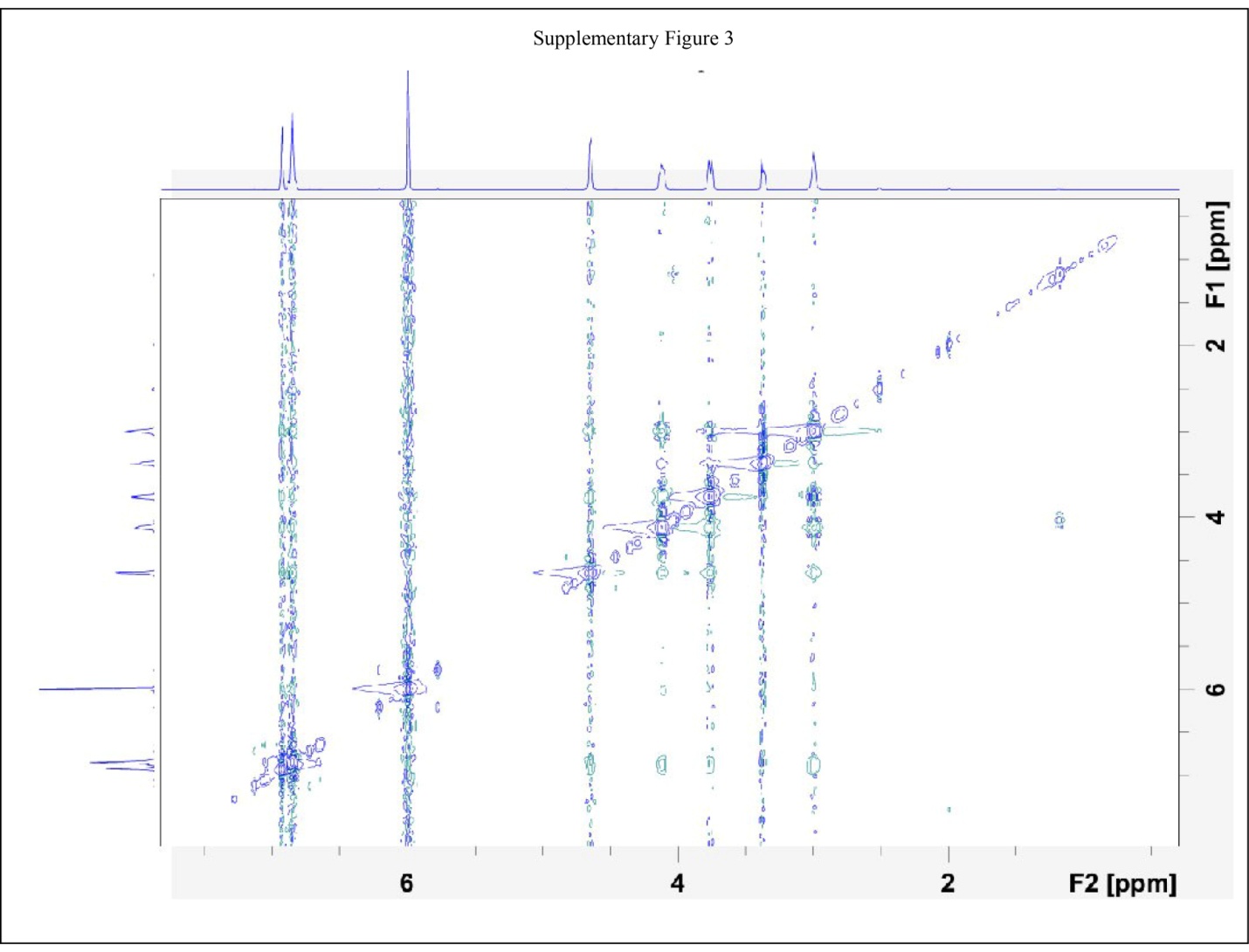


**Supplementary figure 4.** COSY spectra of isolated phyto-component S8 from ethyl acetate fraction of hydromethanolic stem extract of *Zanthoxylum armatum* DC.


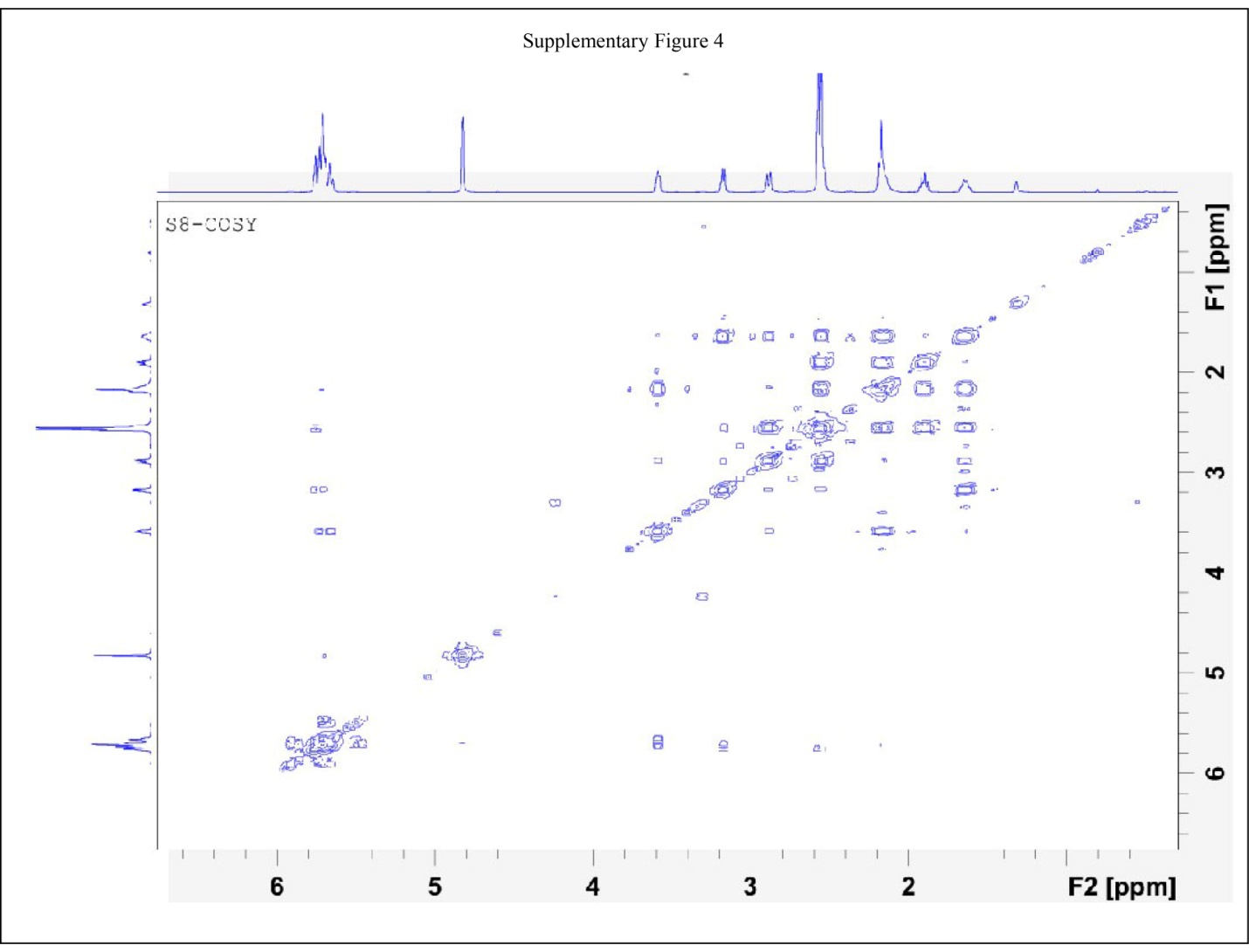


**Supplementary figure 5.** NOESY spectra of isolatedphyto-component S8 from ethyl acetate fraction of hydromethanolic stem extract of *Zanthoxylum armatum* DC.


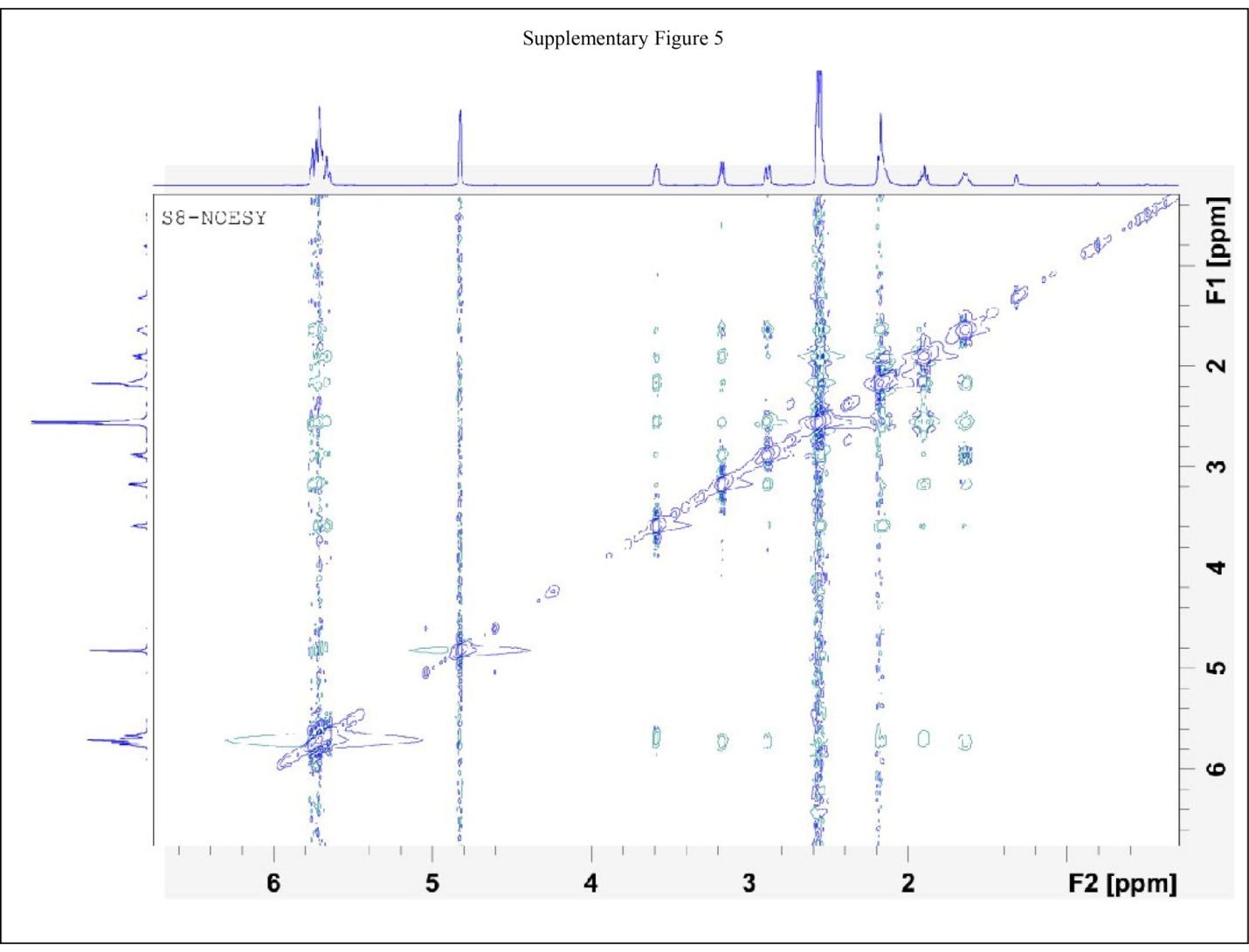


**Supplementary figure 6.** ROSEY spectra of isolatedphyto-component S8 from ethyl acetate fraction of hydromethanolic stem extract of *Zanthoxylum armatum* DC.

**
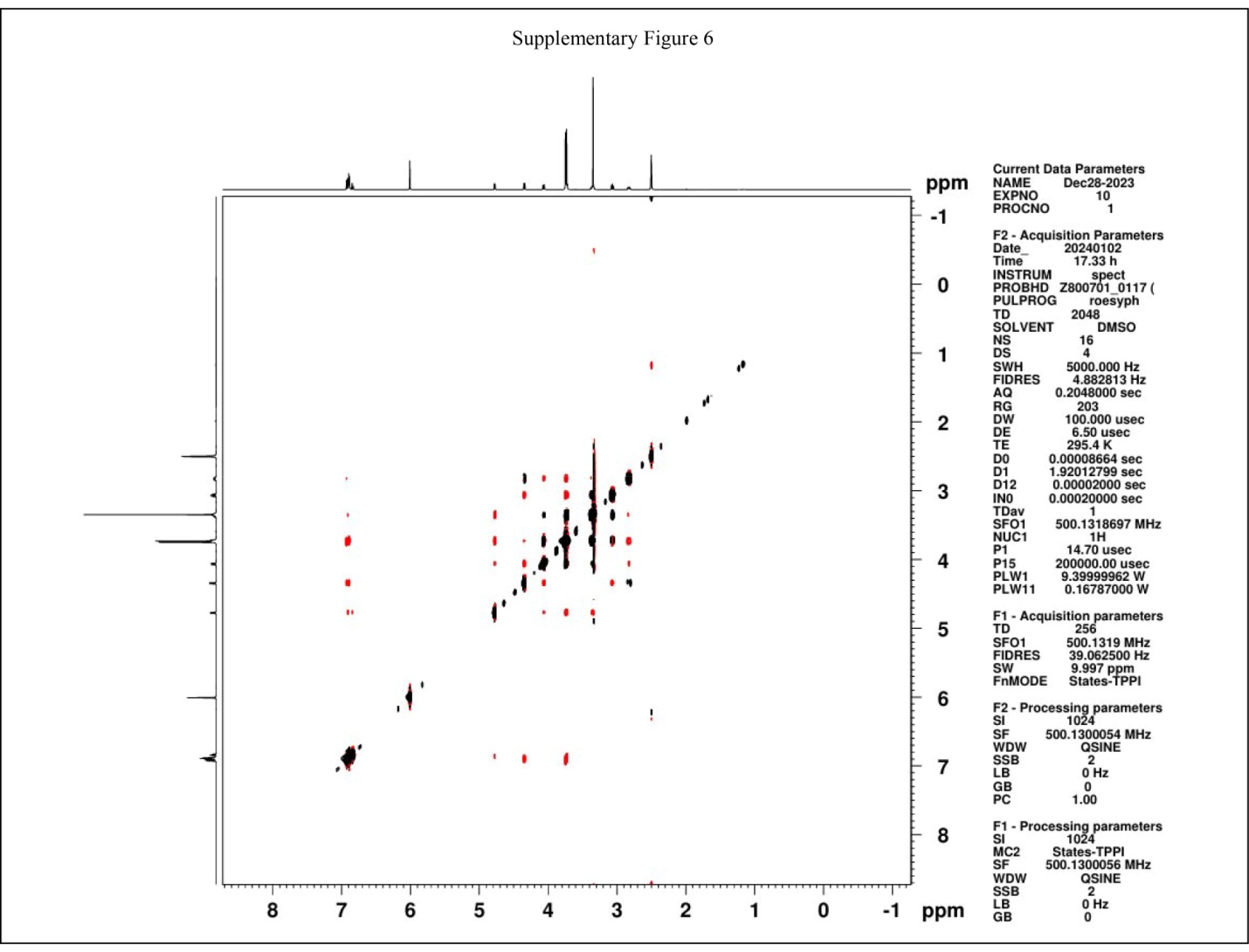
**

**Supplementary figure 7.** DEPT 135 spectra of isolatedphyto-component S8 from ethyl acetate fraction of hydromethanolic stem extract of *Zanthoxylum armatum* DC.


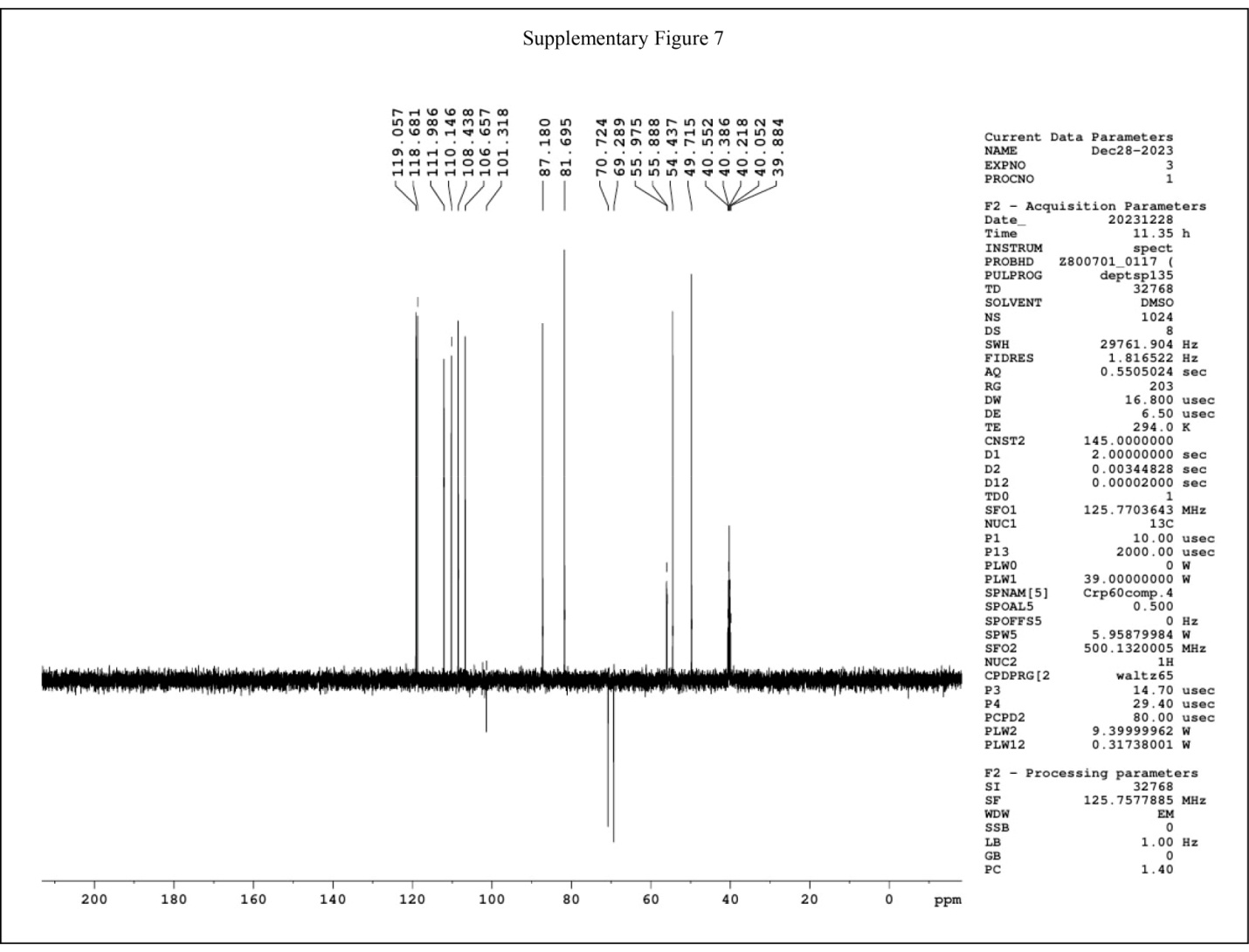


**Supplementary figure 8.** H HMBC spectra of isolatedphyto-component S8 from ethyl acetate fraction of hydromethanolic stem extract of *Zanthoxylum armatum* DC.


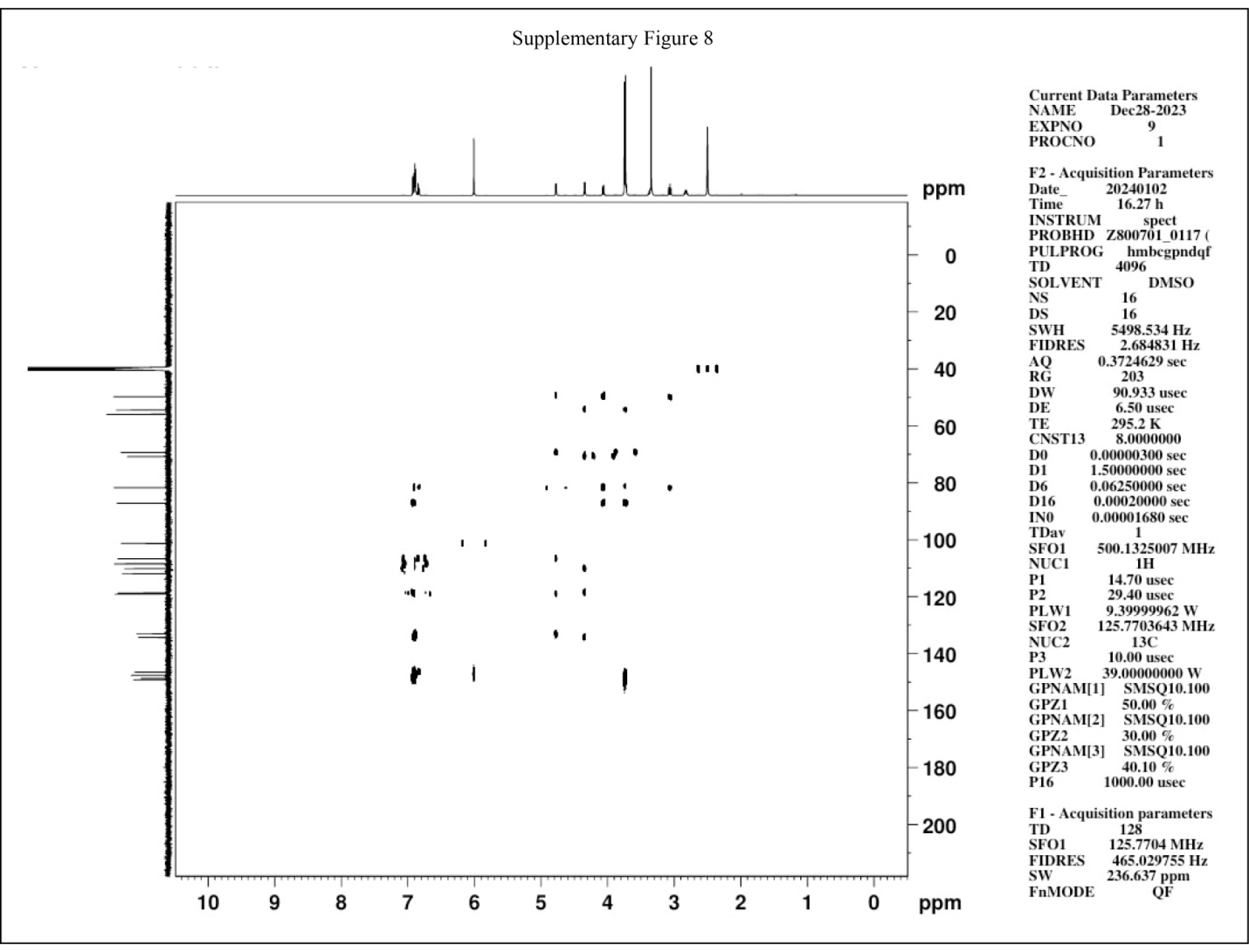


**Supplementary figure 9.** H HMQC spectra of isolatedphyto-component S8 from ethyl acetate fraction of hydromethanolic stem extract of *Zanthoxylum armatum* DC.


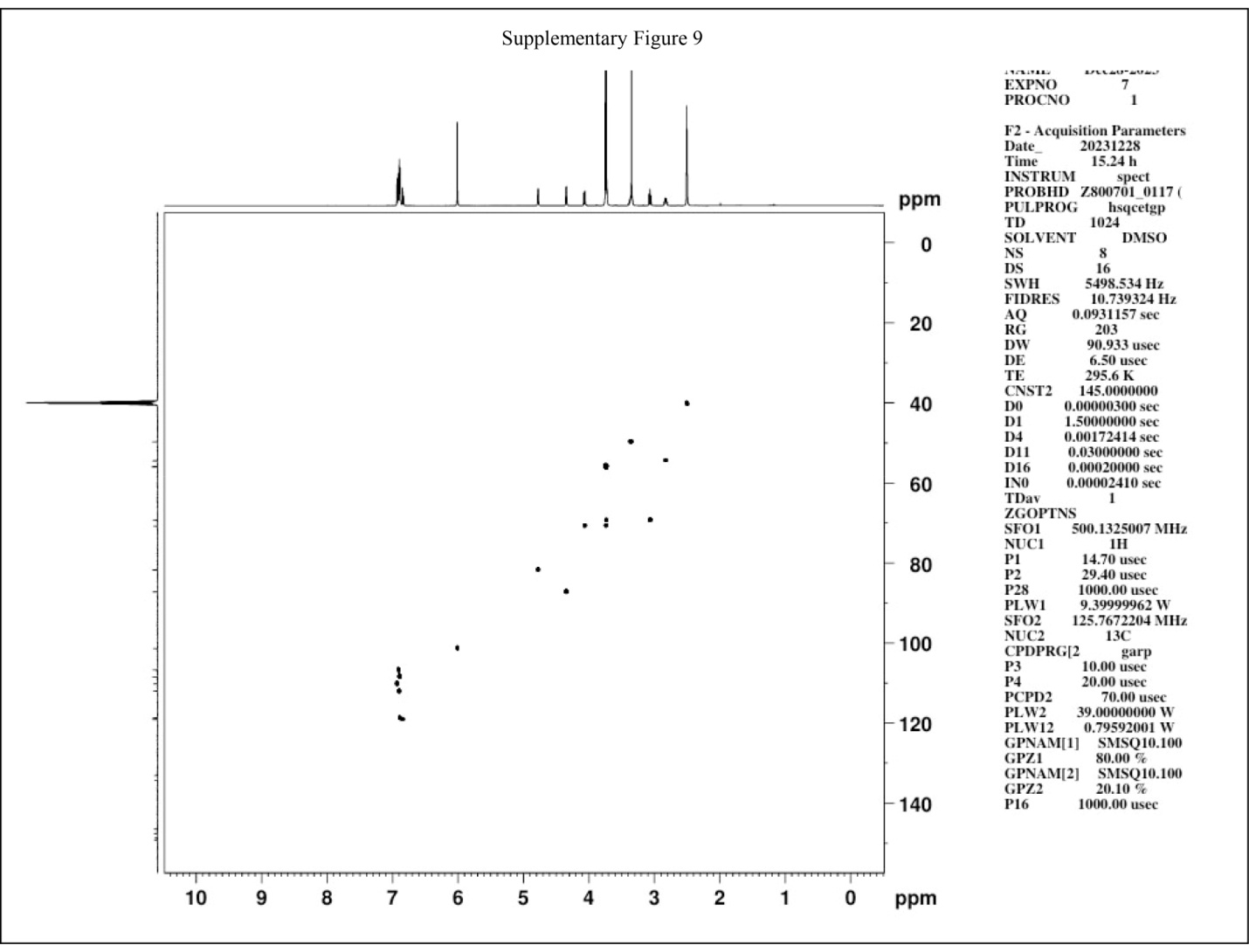

Supplement: Supplementary file 1 [file Supplementaryfile1.docx]
